# Unveiling microbial dynamics: a review of health and immune enhancement in school settings

**DOI:** 10.3389/frmbi.2024.1488702

**Published:** 2024-12-04

**Authors:** Philip Asumang, Richard Ntumi, Francis Dwomoh

**Affiliations:** ^1^ Department of Science, Seventh-Day Adventist College of Education, Agona, Ashanti, Ghana; ^2^ Kwame Nkrumah University of Science and Technology, Kumasi, Ghana; ^3^ School of Medicine, Saint Vincent and the Grenadines, American University of St. Vincent, Kings Town, Saint Vincent and the Grenadines

**Keywords:** microbial dynamics, immune system, health, school environment, human microbiome

## Abstract

This review focuses on the role of microorganisms in promoting health and immune function within school environments. Microbes, including bacteria, viruses, fungi, and other microorganisms, constitute the human microbiome and play a crucial role in various bodily functions and immune system development. The complex interactions between microorganisms and the immune system in schools, where children spend a significant amount of time, are not fully understood. While schools have traditionally emphasized hygiene practices to prevent the spread of infectious diseases, recent research has highlighted the potential consequences of reduced microbial exposure during early life. The “hygiene hypothesis” suggests that limited exposure to microbes in infancy may increase the risk of allergies, asthma, and autoimmune diseases in adulthood. This paper explores the microbial diversity found in schools, the benefits of exposure to different microorganisms, and the implications of hygiene practices on immune system development. It also examines current research on microbial intervention strategies and their potential to influence overall health in schools. Understanding the role of microbes in school environments has implications for public health policies and educational practices, aiming to create healthier and more conducive learning environments for the younger generation. By comprehensively exploring this topic, this review contributes to a broader understanding of the significance of microbes in promoting health and immune function in school settings and its relevance to future health research.

## Graphical Abstract

A system dynamics approach to understanding the health and immune enhancement in school settings.

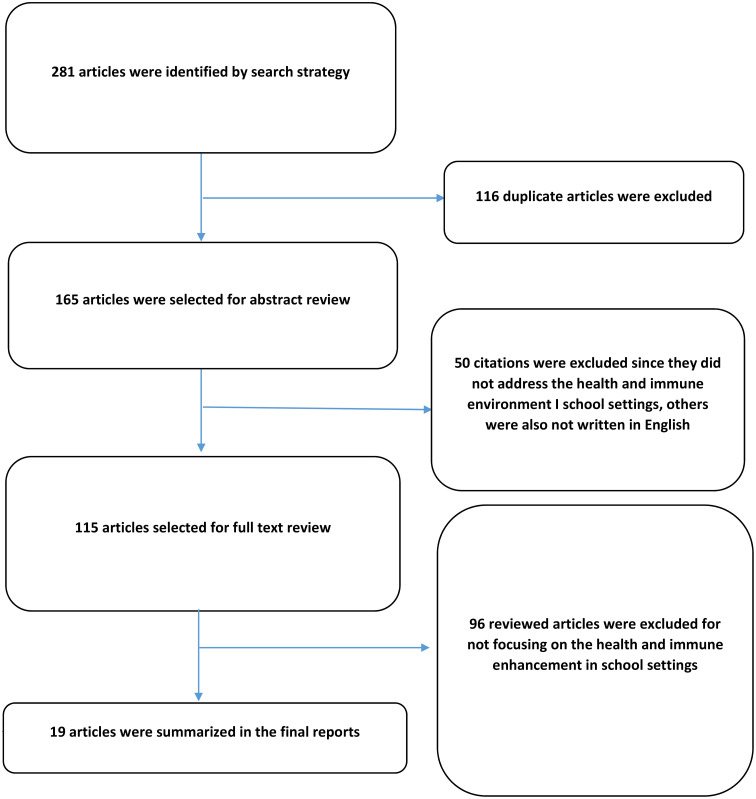

## Introduction

In recent times, there has been a growing focus and research interest in understanding the influence of microorganisms on human health and immune system functionality (Postler and Ghosh, 2017). The human microbiome, a dynamic environment comprising bacteria, viruses, fungi, and other microorganisms, is crucial for supporting multiple bodily processes such as digestion, metabolism, and the maturation of the immune system (Morar and Bohannan, 2019). However, the intricate relationship between microorganisms and the immune system within school environments, where children spend a significant amount of time, is not fully understood (Gensollen et al., 2016). Schools, bustling with students and faculty from diverse backgrounds, harbor a plethora of microbial communities that contribute to the shared environment. This exposure to a varied diversity of microbes during a crucial period of immune system development can have profound effects. The immune system, highly malleable and sensitive to environmental influences, undergoes crucial developmental stages during childhood (Goenka and Kollmann, 2015). As children encounter various microbes in their environment, their immune system adapts and learn to distinguish between innocuous substances and potential dangers. This learning process is vital for the development of a robust and well-balanced immune system that can defend against infections while avoiding detrimental inflammatory responses. Historically, schools have emphasized hygiene practices as a means of averting the spread of infectious diseases (Fee, 2016). Nevertheless, recent research has shed light on the potential outcomes of decreased exposure to microbes during infancy, emphasizing the significance of maintaining proper hygiene practices (Finlay et al., 2021; Renz et al., 2017; Stiemsma et al., 2015). hygiene hypothesis suggests that limited exposure to microbes during infancy may increase the risk of allergies, asthma, and autoimmune diseases in adulthood, challenging conventional wisdom regarding cleanliness.

The early colonization of an infant’s gut by microorganisms plays a crucial role in their overall health and immune development. This process is influenced by various factors, including maternal microbiota, mode of delivery, and feeding practices.

Maternal microbiota significantly influences infant health right from pregnancy. Research indicates that the composition of a mother’s gut, oral, and vaginal microbiota can affect fetal development through vertical transmission during childbirth. The gut microbiota of mothers undergoes changes due to diet, antibiotic use, and other environmental factors, which in turn shape the microbial landscape of the newborn (Dzidic et al., 2018; Yao et al., 2021). For instance, beneficial microbes such as *Lactobacilli* and *Bifidobacteria* are essential for establishing a healthy gut microbiome in infants, contributing to immune system development and metabolic programming (Obermajer et al., 2017; Yao et al., 2021).

The mode of delivery is a critical determinant of an infant’s initial microbial exposure. Infants born via vaginal delivery are exposed to their mother’s vaginal and gut microbiota, which helps establish a diverse microbial community. Conversely, caesarean section deliveries often result in a less diverse microbiome due to reduced exposure to maternal bacteria (Dunn et al., 2017). This lack of diversity has been linked to increased risks for conditions such as asthma, allergies, and obesity later in life (Dunn et al., 2017).

Breastfeeding is another vital factor influencing the infant microbiome. Breast milk contains prebiotics that promote the growth of beneficial bacteria like *Bifidobacterium* while inhibiting pathogens. Studies show that infants who are exclusively breastfed develop healthier gut microbiomes compared to those who receive formula supplementation (Obermajer et al., 2017; Yao et al., 2021). Early introduction of solid foods or formula can disrupt this delicate balance, leading to potential long-term health issues (Obermajer et al., 2017).

Emerging research also suggests that the paternal gut microbiome may affect infant health. Disruptions in the father’s gut microbiota have been correlated with negative outcomes in offspring, such as low birth weight and stunted growth. Although the mechanisms are not fully understood, they may involve hormonal changes and nutrient supply alterations during pregnancy (Ishimwe, 2021).

The implications of early microbial colonization extend into adulthood. An imbalance in microbial populations during infancy has been associated with various chronic conditions later in life, including metabolic disorders and autoimmune diseases (Dunn et al., 2017; Obermajer et al., 2017). The establishment of a healthy microbiome is thus critical for long-term health outcomes.

While the role of microorganisms in enhancing infant health and immune development is well-documented, it is essential to recognize the limitations and potential risks associated with microbial exposure. A balanced perspective that addresses these limitations while emphasizing the beneficial effects provide a comprehensive understanding of the review. Microorganisms play a critical role in shaping an infant’s gut microbiome, which is essential for their overall health. The beneficial effects include: Early microbial colonization helps train the immune system, reducing the risk of allergies and autoimmune diseases later in life (Renz and Skevaki, 2021). Beneficial bacteria such as *Bifidobacteria* enhance nutrient absorption and synthesis of essential vitamins, contributing to better growth and development (Bakshi et al., 2023). A diverse microbiome can outcompete harmful bacteria, providing a protective barrier against infections (Chiu et al., 2017). The gut microbiome influences metabolic processes, potentially reducing the risk of obesity and related metabolic disorders (Vallianou et al., 2019).

Despite these benefits, several limitations must be addressed to ensure optimal health outcomes: An imbalance in microbial populations often referred to as dysbiosis can lead to health complications such as gastrointestinal disorders and increased susceptibility to infections (de Oliveira et al., 2021). Infants born via caesarean section may miss out on beneficial maternal microbes, leading to a less diverse gut microbiome. This lack of diversity has been associated with higher risks for conditions like asthma and obesity (Michalovich et al., 2019). While breastfeeding is beneficial for establishing a healthy microbiome, early introduction of solid foods or formula can disrupt this balance. Parents should be educated about optimal feeding practices to support microbial health (Smarta, 2023). Factors such as antibiotic use during pregnancy or infancy can adversely affect microbial colonization and diversity. Awareness around judicious antibiotic use is crucial for maintaining a healthy microbiome (Dhingra et al., 2020).

This paper purpose is to explore the role of microbes in promoting health and immune function in school environments. We will delve into the microbial diversity found in schools, the potential benefits of exposure to various microorganisms, and the implications of hygiene practices on immune system development. Additionally, we will examine current research on microbial intervention strategies and their potential to influence overall health in schools. Comprehending the intricate relationships among microorganisms and the immune system in school settings has implications beyond the immediate well-being of students and staff (National Academies of Sciences & Medicine, 2017). It will inform public health policies and educational practices, aiming to create healthier and more conducive learning environments for the younger generation. By comprehensively exploring this topic, we aim to enhance comprehension regarding how microbes support health and immune function within school environments, thereby highlighting their importance for future health studies.

## The general overview of microbes

Microorganisms, often referred to as microbes, are microscopic living entities that cannot be observed without the aid of a microscope (Ingraham, 2018). They are incredibly diverse and include bacteria, archaea, fungi, protists, and viruses. Found in almost every environment on Earth, microbes play essential roles in various biological processes, influencing ecosystems and life as a whole (Prosser et al., 2007).

### Bacteria

**Figure 1:**
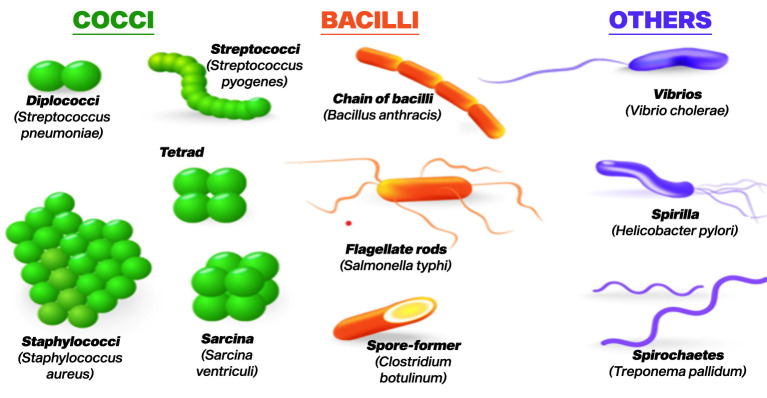
Various shapes of bacteria (Koca, 2024).

Bacteria represent one of the most plentiful and varied categories of microorganisms found on the planet (Flemming and Wuertz, 2019). Bacteria are unicellular prokaryotic organisms, lacking a distinct nucleus and other membrane-bound structures typically seen in eukaryotic cells. Despite their uncomplicated organization, bacteria exhibit impressive adaptability and versatility, flourishing in nearly every conceivable environment (Leadbetter and Poindexter, 2013). Bacteria exist in a variety of shapes, including spheres (cocci), rods (bacilli), and spirals (spirilla) as in **Figure 1** (Koca, 2024). Some bacteria have a firm cell wall, while others have a flexible cell wall or lack one altogether (Huang et al., 2008). Cell wall is essential for maintaining the shape of bacteria and protecting them from environmental stresses. Additionally, bacteria may possess flagella for movement and pili for attachment to surfaces or other cells.

Bacteria can be found in virtually every habitat on Earth, from deep ocean trenches to high-altitude mountains, from the frozen polar regions to scorching hot springs (Jacobsen and Dangles, 2017). They can be found in various environments such as soil, water, air, and even within the organisms of plants and animals, including humans. Bacteria often form complex communities called biofilms, where they adhere to surfaces and interrelate with each other in a accommodating manner. Bacteria are vital for the process of recycling nutrients and breaking down organic matter. Bacteria serve as key decomposers, breaking down deceased organic material and reintroducing nutrients into the ecosystem’s cycle (Gupta et al., 2017). Without bacteria, essential elements such as carbon, nitrogen, and phosphorus would be trapped in dead organic material and unavailable for use by other organisms.

Bacteria form various symbiotic relationships with other organisms (Douglas, 2021). Some bacteria are mutualistic, meaning both the bacterium and its host benefit from the association. As an illustration, nitrogen-fixing bacteria inhabit the root nodules of leguminous plants, transforming atmospheric nitrogen into a form that these plants can utilize. This provides a vital nutrient for the plant, while the bacteria receive nutrients from the plant in return. Bacteria also play a critical role in the digestive systems of animals, helping to break down complex compounds and aiding in digestion (Zhang et al., 2015). Bacteria have immense biotechnological importance. They are used in the production of food products like yogurt, cheese, and fermented beverages (Irkin, 2019). Bacteria are utilized in the manufacturing of antibiotics, enzymes, and a variety of biofuels. Genetic engineering techniques use bacteria as host organisms to produce recombinant proteins and other biotechnological products. While many bacteria are beneficial or neutral, Certain bacteria are pathogenic and have the potential to induce illnesses in humans, animals, and plants. These pathogenic bacteria can yield toxins or invade host tissues, leading to illnesses ranging from mild infections to severe and life-threatening conditions. One major concern in the medical field is the rise of antibiotic-resistant bacteria (Serwecińska, 2020). The excessive and improper use of antibiotics has resulted in the emergence of bacterial varieties that can withstand numerous commonly prescribed medications. This poses a significant challenge for healthcare professionals in treating bacterial infections. Bacteria are incredibly diverse and versatile microorganisms that play vital roles in numerous ecological processes and have substantial impacts on human life and industry. Their study is critical for understanding both the natural world and the development of biotechnological advancements and medical treatments.

It was noted that frequently touched surfaces like keyboards and faucet handles had high bacterial counts (Alkhamis and Noweir, 2018; Sifuentes et al., 2017). Similarly, research found elevated levels of Staphylococcus aureus in high-traffic areas of schools (Małecka-Adamowicz et al., 2019). Furthermore, Alexandrovna and Nikolayevich’s study reported significant bacterial contamination on school desks, with Staphylococcus spp. being frequently detected (Alexandrovna and Nikolayevich, 2023). These findings underscore the importance of maintaining hygiene in educational settings to mitigate health risks associated with microbial exposure.

However, while the prevalence of bacteria is concerning, it is also essential to consider the role of proper hygiene practices in reducing these microbial loads, as demonstrated by intervention studies that showed decreased absenteeism due to illness in cleaner classrooms.

### Fungi

**Figure 2:**
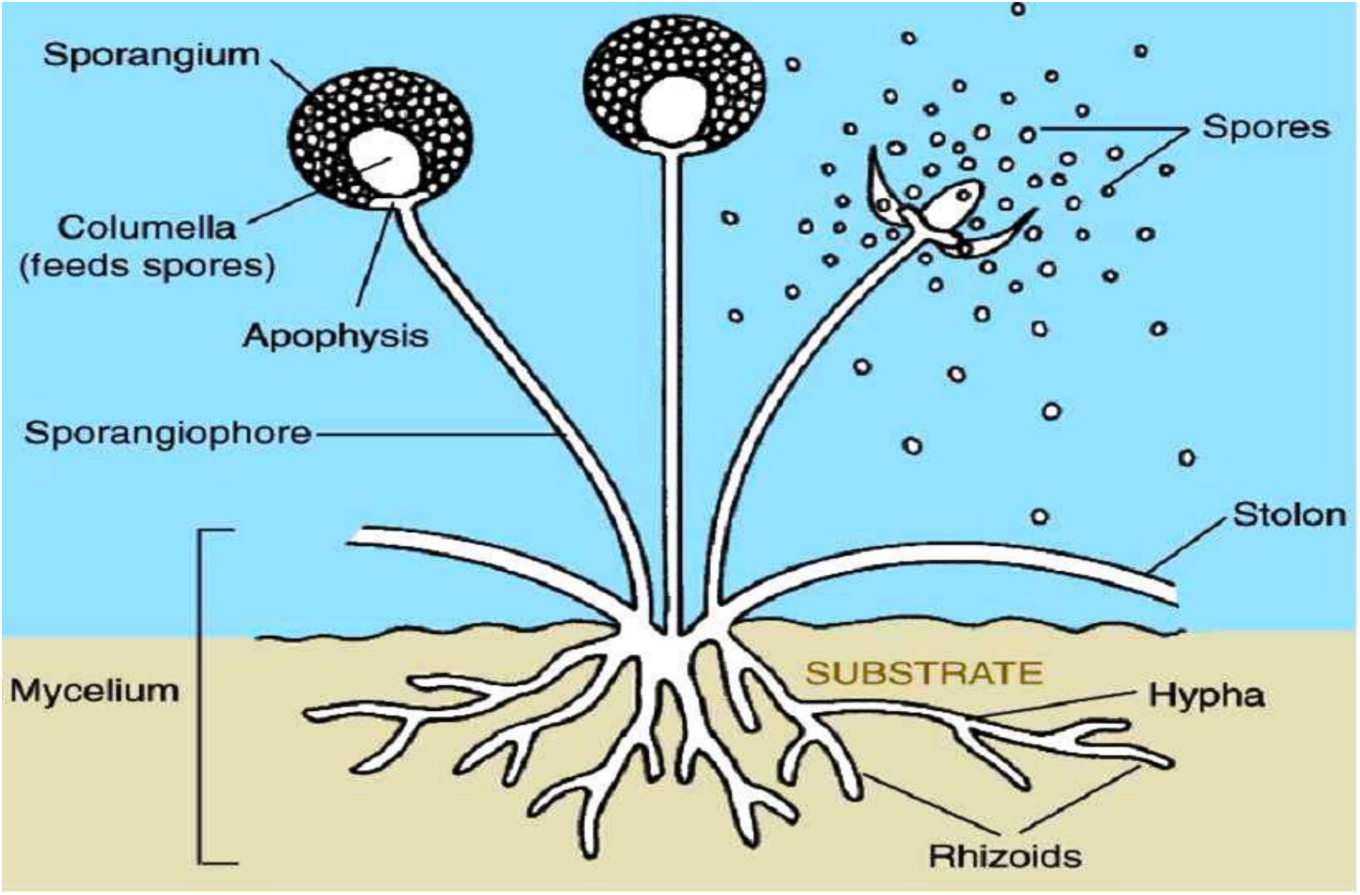
Basic structure of fungi (Senanayake et al., 2020).

Fungi represent a varied collection of eukaryotic microorganisms that fulfill essential functions within ecosystems and exert notable influences on various aspects of life (Grossart et al., 2019). They exhibit a wide range of forms and functions, and their presence can be found in almost every environment on Earth. From **Figure 2** (Senanayake et al., 2020), fungi are characterized by their unique body structure, which typically consists of thread-like structures called hyphae (Mukherjee and Ghorai, 2023). These hyphae intertwine to create a sophisticated network referred to as mycelium. The mycelium acts as the central structure of the fungus and is often hidden within the substrate, while reproductive structures, such as mushrooms or fruiting bodies, are visible above the surface.

Fungi are primarily decomposers, playing a critical role in breaking down dead organic matter (Frey, 2019). They release enzymes into their environment, which decompose complex organic substances such as cellulose and lignin into smaller molecules that can be assimilated as nutrients. This process of decomposition is essential for recycling nutrients back into the ecosystem, making fungi critical players in nutrient cycling. Fungi form various symbiotic relationships with other organisms (Martin et al., 2017). One well-known example is mycorrhizal fungi, which establish mutualistic partnerships with the roots of most plants. In mycorrhizal associations, the fungi help plants absorb essential nutrients, such as nitrogen and phosphorus, while the plant offers the fungus with sugars produced through photosynthesis. This symbiosis benefits both the fungi and the plants involved.

Fungi play a fundamental role in maintaining ecosystem balance (Dighton, 2018). As decomposers, they break down dead organic matter, releasing nutrients which can be utilized by other living organisms. Additionally, fungi serve as food sources for various animals and play a vital role in shaping plant communities through their interactions with plants. Fungi hold economic significance in various ways. Many fungi are edible and provide a source of nutrition for humans, with mushrooms being the most well-known example (El-Ramady et al., 2022). Fungi are additionally employed in crafting a variety of fermented foodstuffs and drinks, including bread, cheese, beer, and wine. Furthermore, certain fungi have biotechnological applications in industries like agriculture, pharmaceuticals, and biofuels.

For centuries, fungi have been a crucial cornerstone in the development of medicinal treatments. Antibiotics like penicillin and cyclosporine were discovered from fungi and have revolutionized medicine (Durand et al., 2019). Fungi also produce various enzymes and bioactive compounds that have medicinal applications and potential treatments for various diseases. While many fungi are beneficial or neutral, some can be pathogenic and cause diseases in plants, animals, and humans (Köhler et al., 2015). Fungal infections, known as mycoses, can range from mild superficial infections like athlete’s foot to severe systemic infections that may be life-threatening, especially in immunocompromised individuals. Fungi are a fascinating and varied group of microorganisms that play essential roles in ecosystems, agriculture, medicine, and various industries (Buckley, 2020). Their ability to decompose organic matter, form symbiotic relationships, and provide valuable resources and medicines makes them critical to the functioning of the natural world and human society.

A study conducted in Maltese schools identified high levels of fungi from the *Penicillium/Aspergillus/Paecilomyces/Variotii* group, which were linked to poor indoor air quality and respiratory health issues among students (Fsadni et al., 2017). The presence of these fungi was associated with increased asthma and allergic reactions, highlighting the importance of maintaining clean and well-ventilated school environments to protect student health (Nath et al., 2023).

Research assessing indoor microbial pollutants in schools found that elevated levels of *Aspergillus versicolor* were present in several classrooms, exceeding recommended thresholds (Kumar et al., 2022). This study established a correlation between fungal exposure and respiratory problems among children, emphasizing the need for effective cleaning protocols and environmental management to reduce allergenic exposures in educational settings.

### Protists

**Figure 3:**
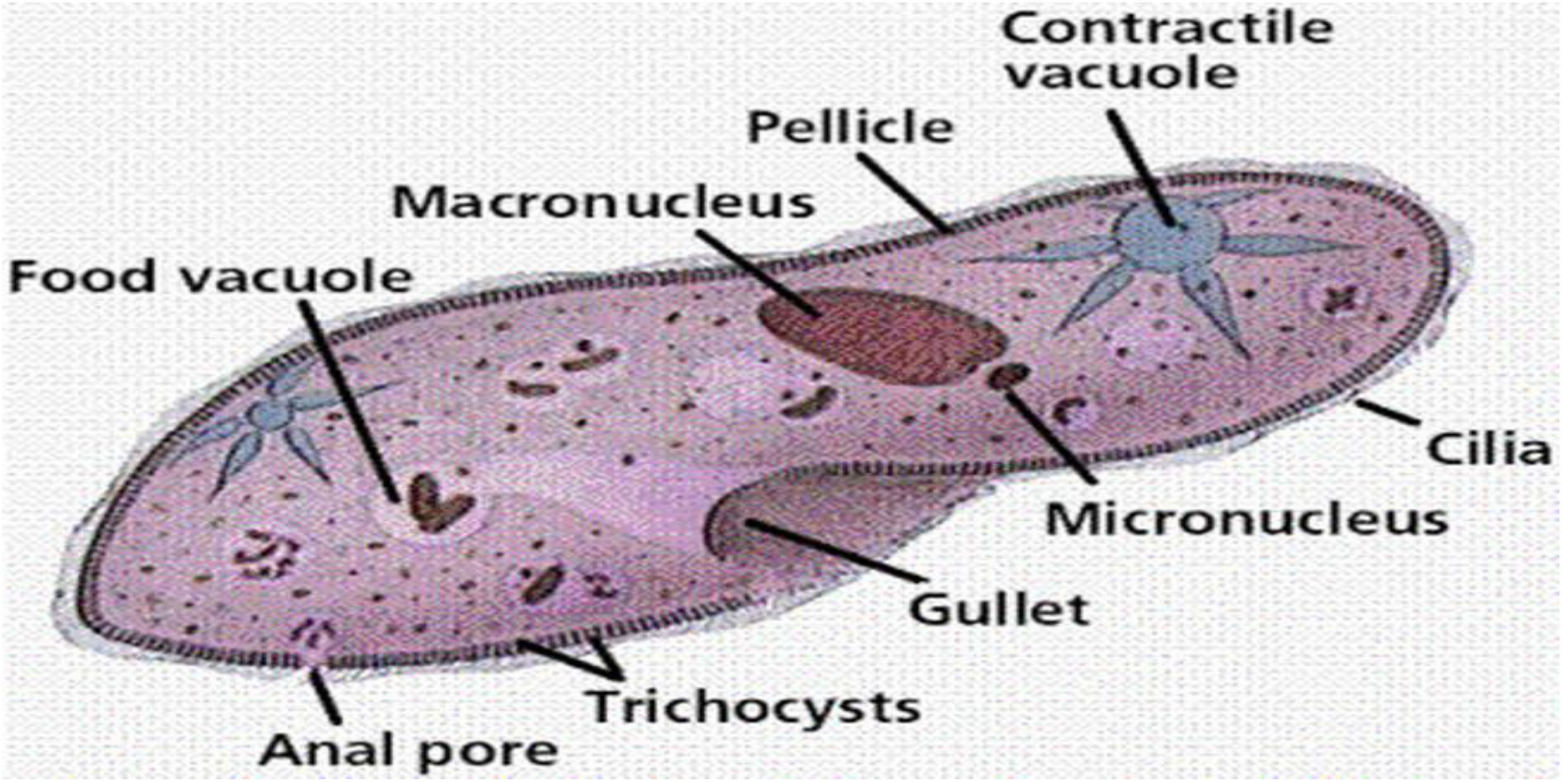
Basic structure of a Protists (Soares et al., 2019).

Protists encompass a broad range of eukaryotic microorganisms that defy classification within the traditional categories of plants, animals, or fungi (Verma, 2021). They are single-celled or multicellular organisms with an extensive variety of shapes, sizes, and life cycles (**Figure 3**) (Soares et al., 2019). Protists inhabit a variety of environments, such as freshwater and marine ecosystems, soil, and the internal systems of plants and animals (Singer et al., 2021). Protists exhibit diverse nutritional strategies (Liu et al., 2016). They can be photosynthetic, like algae, which produce their food using sunlight and carbon dioxide. Others are heterotrophic, feeding on organic matter or other organisms. Approximately protists are mixotrophic, merging both photosynthetic and heterotrophic modes of nutrition. Protists play crucial ecological roles in various ecosystems. Photosynthetic protists, like phytoplankton in the ocean, are primary producers, forming the basis of the food chain and providing oxygen through photosynthesis (Decelle et al., 2015). They also contribute to carbon and nutrient cycling in aquatic environments.

As primary producers or prey for other organisms, protists serve as an essential link in food chains (Weitere et al., 2018). Protists serve as an important food source for numerous aquatic creatures, including zooplankton, small fish, and invertebrates. Protists have the capability to establish symbiotic associations with other organisms. For example, some protists form mutualistic partnerships with corals, providing them with nutrients through photosynthesis while receiving shelter and protection in return. Although the majority of protists are harmless or even beneficial, certain species have the Capability to cause diseases in humans, animals, and plants (Geisen et al., 2018). Malaria, caused by the protist Plasmodium, is one of the most well-known examples of a disease caused by a protist. Classifying and studying protists can be challenging due to their incredible diversity and complex life cycles. Traditional taxonomic methods are often insufficient, and molecular techniques have become increasingly important in understanding their evolutionary relationships. Protists are a varied and fascinating group of eukaryotic microorganisms with significant ecological importance (Caron and Hu, 2019). They inhabit a wide range of habitats and play various roles in ecosystems, from being primary producers to forming symbiotic relationships. While some protists are pathogens, Numerous other protists play a critical role in maintaining ecosystem equilibrium and contribute significantly to overall ecological health of the planet (Zwair, 2023). Understanding and studying protists are essential for advancing our knowledge of biodiversity, evolution, and the functioning of ecosystems.

A study highlighted the presence of protozoan parasites such as *Giardia lamblia* and *Cryptosporidium* in school water supplies and surfaces, which can lead to gastrointestinal illnesses among students (Hajare et al., 2022). The research emphasized the need for regular testing and monitoring of water quality in schools to prevent outbreaks linked to protozoan contamination.

Another investigation assessed the health effects of protozoan infections in school-aged children, linking exposure to contaminated water sources with increased rates of diarrhea and absenteeism from school (Anyango, 2019). This study underscored the importance of ensuring safe drinking water and sanitation facilities within educational institutions to protect children’s health.

### Viruses

**Figure 4:**
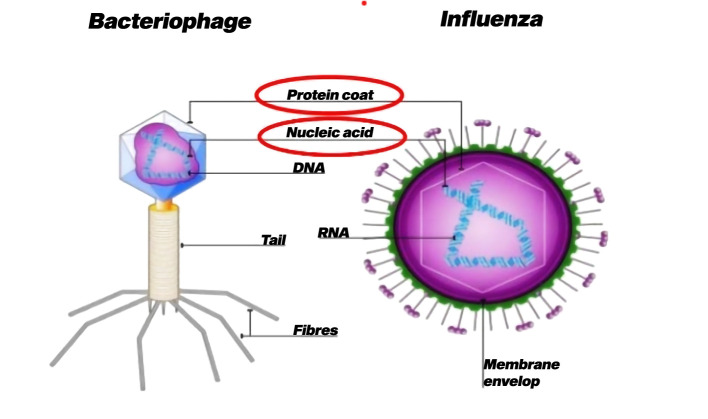
Basic structure of virus (Ackermann et al., 2021).

Viruses are unique and intriguing entities that represent a distinct group of microorganisms (Colson et al., 2018). Protists do not fit neatly into any of the conventional biological kingdoms due to their deviation from typical characteristics exhibited by other living organisms. Viruses are categorized as obligate intracellular parasites because they rely on a host cell to replicate and fulfill their life cycle (Ryu, 2017). Viruses generally contain a small amount of genetic material, either DNA or RNA, enclosed in a protein coat called a capsid (**Figure 4**) (Ackermann et al., 2021). Some viruses may additionally have additional structures, such as an outer lipid envelope obtained from the host cell membrane (Ketter and Randall, 2019). Unlike living cells, viruses lack the cellular machinery required for energy production, metabolism, or protein synthesis. Viruses lack the ability to carry out essential life processes independently and entirely rely on host cells for reproduction.

The replication of viruses involves infecting a host cell and taking control of its cellular machinery to produce multiple copies of the virus (Rampersad and Tennant, 2018). This often leads to the destruction of the host cell and the issue of new viral particles, which can go on to infect other cells. Viruses demonstrate a remarkable specificity for their host cells, as each virus has the capability to infect only particular types of cells within specific species. For example, a virus that causes a cold in humans cannot infect plants or animals. While many viruses are harmless to their hosts, some can cause diseases in numerous organisms, including humans, animals, plants, and even other microorganisms (Sabirovna, 2021). Viral diseases vary in severity, spanning from mild infections to severe and potentially life-threatening illnesses. Vaccines have been developed for several viral diseases, helping to prevent their spread and reduce the severity of infections.

Viruses have the potential to emerge from animal reservoirs and infect new hosts, leading to outbreaks and pandemics. Examples include the emergence of the Ebola virus, Zika virus and SARS-CoV-2 (the virus causing COVID-19) (Ortiz-Prado et al., 2020). Some viruses have been harnessed for beneficial purposes in biotechnology. Viral vectors are used to deliver genetic material into cells for gene therapy and genetic engineering applications. Despite their significant impact on human health and biotechnology, viruses exist in a state between living and non-living entities (Dupré and Guttinger, 2016). They do not meet the criteria for life as they cannot reproduce, metabolize, or respond to stimuli on their own. However, they can undergo evolution through mutation and natural selection. Viruses are unique and fascinating entities that challenge our understanding of life. As obligate intracellular parasites, they rely entirely on host cells for replication. Understanding viruses is essential for managing infectious diseases, developing vaccines, and advancing our knowledge of molecular biology and evolution (Dupré and Guttinger, 2016).

A study by Yale researchers focused on microbial communities on school desks, revealing that viruses and bacteria predominantly originated from students themselves (Kwam, 2018). The findings indicated that even after cleaning, microbial populations returned quickly, suggesting that desks serve as significant vehicles for pathogen transmission among students. This has implications for public health, particularly during outbreaks or for children with compromised immune systems.

Research conducted by the University of Arizona demonstrated that proper cleaning and disinfection protocols significantly reduced norovirus contamination on surfaces within classrooms (Abney, 2022). The study found that implementing hand sanitizers and disinfecting key surfaces led to a more than 50% reduction in norovirus occurrence, thereby decreasing student absenteeism due to gastrointestinal illness.

Microbes have significant impacts on the environment and human society. They are vital for nutrient cycling, decomposition, and maintaining ecological balance (Gupta et al., 2017). Additionally, microbes have numerous biotechnological applications, such as food production through fermentation, medicine production like antibiotics, and biofuel generation (Gupta et al., 2017). They also have a crucial impact on human health, which are present in the human microbiome, supporting digestion, immune function, and overall well-being. However, some microbes can be harmful, causing infectious diseases in humans, animals, and plants. Comprehending the mechanisms of pathogenic microorganisms is vital for preventing, diagnosing, and treating diseases.

## The human microbiome and immune system

**Figure 5:**
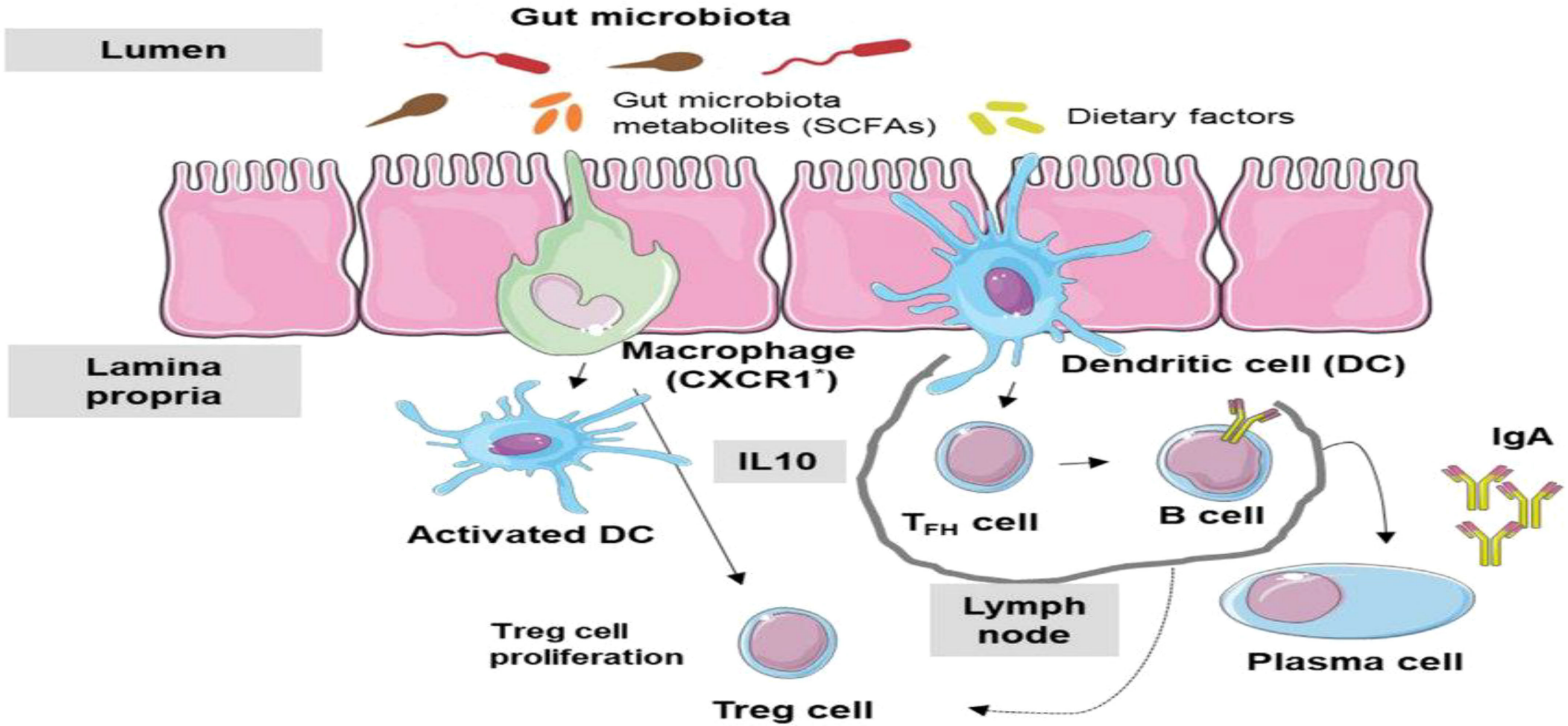
Microbiome-Immune System Interactions: immune system responds to microbiome dysbiosis (Cheng et al., 2019).

Higher organisms possess both a general and increasingly specialized immune system from birth. These immune mechanisms are designed to assist the body in defending against infections (Lambring et al., 2019). A variety of internal and external factors can impact the immune system, and its responses are tailored to suit each unique circumstance. Alongside the innate and adaptive immune systems, the human body contains interconnected systems (**Figure 5**). Advances in understanding how these systems interact with the immune system have led to new research directions, particularly in microbiome studies. Over the past two decades, interest in studying the microbiome has surged, largely due to the NIH’s support for the Human Microbiome Project (HMP) initiated in 2007. Numerous studies have focused on characterizing and understanding the functions of the microbiome, as well as its intricate interactions with the rest of the body. Dysfunctions in the relationship between the microbiome and the host have been linked to a variety of diseases, including cancers, metabolic disorders, autoimmune conditions, and infectious diseases (Lambring et al., 2019). A deeper comprehension of how the microbiome interacts with the host in the context of diseases is imperative for grasping the inferences of microbiome dysfunction and exploring potential utilization of microbiota in disease prevention.

## Microbial community and structure

Microbial communities in educational settings significantly influence the health and immunological responses of pupils. Comprehending the makeup and behavior of these microbial communities is essential for evaluating their potential effects on public health.

Bacterial populations in classrooms are affected by human activities and environmental variables (Flandroy et al., 2018). A study of classroom floor dust in public primary schools highlighted the significant influence of outside bacterial sources on microbial composition, with human inhabitants also contributing notably (Park et al., 2021). Utilizing 16S rRNA sequencing, researchers identified a diverse array of bacterial species, revealing that the classroom microbiome differs from typical home environments, which are more dominated by human-associated bacteria. The study identified 29 phyla and over 2,000 species, with Proteobacteria being the most diverse and Firmicutes the most abundant (Park et al., 2021). Outdoor-associated genera were more prevalent in classrooms, contrasting with home dust, which is richer in human-associated bacteria (Park et al., 2021). Human presence significantly affects indoor microbial concentrations, with occupancy leading to elevated levels of bacteria and fungi (Hospodsky et al., 2014). While human-associated taxa were present, outdoor sources often dominated the microbial composition in well-ventilated spaces (Adams et al., 2015). The distinct microbial profiles in classrooms may have different health implications for students and staff compared to home environments, necessitating further research into exposure effects (Täubel and Leppänen, 2017).

Also, fungi play significant role in the microbial population within educational institutions, particularly in Malta, where studies have identified high levels of fungi from the Penicillium/Aspergillus/Paecilomyces group. These fungi are linked to respiratory issues among students, highlighting the importance of understanding their impact on indoor air quality and health. Research indicated that schools, especially primary institutions, exhibit elevated concentrations of fungi, with the Penicillium/Aspergillus/Paecilomyces group being predominant (Fsadni et al., 2017). A study found that the median indoor concentration of these fungi was significantly higher than other microbial contaminants, correlating with respiratory health issues in children (Fsadni et al., 2017). Exposure to these fungal spores is associated with increased asthma symptoms, particularly in sensitized children. For instance, children exposed to high levels of Alternaria experienced significantly more asthma symptom days (Baxi et al., 2019; Rathkopf, 2020). The presence of mycotoxins and volatile organic compounds released by fungi can induce allergic reactions and exacerbate respiratory conditions (Upadhyay, 2023). The indoor air quality in schools is influenced by various factors, including cleaning protocols and classroom characteristics, which can either mitigate or exacerbate fungal exposure (Fsadni et al., 2017). Studies emphasize the need for improved management strategies to reduce fungal contamination in educational environments (Upadhyay, 2023)

The investigation of viral communities in educational environments revealed significant insights into pathogen transmission dynamics. Research indicated that viruses on surfaces, such as school desks, primarily originate from human contact, and despite cleaning efforts, these viral populations rapidly re-establish themselves (Adams et al., 2021; Täubel et al., 2024). This highlights the role of educational settings as critical vectors for viral transmission among students. Viruses found on surfaces in schools are largely derived from human interactions, emphasizing the need for effective hygiene practices (Du et al., 2023). Studies showed that even with regular cleaning, viral communities can quickly rebound, suggesting that standard cleaning protocols may be insufficient (Górny et al., 2022; Withey, 2024). The presence of viruses on frequently touched surfaces poses a risk for community-acquired infections, necessitating targeted disinfection strategies (Shicong et al., 2022). Evidence from outbreaks indicated that environmental contamination in schools can lead to significant viral loads, underscoring the importance of monitoring and disinfecting high-risk areas (Liu et al., 2021).

Although infrequently discussed, protozoan contamination can adversely affect student health. A study examining water quality in educational institutions identified the presence of protozoan parasites, including *Giardia lamblia* and Cryptosporidium, in drinking water sources (Ligda et al., 2020; Murugesan et al., 2017). These microbes can cause gastrointestinal problems in students, highlighting the necessity of routine water quality assessments and sanitation protocols (Devane, 2016).

The microbial community composition in educational environments is influenced by a complex interaction between human behavior and environmental conditions. Bacteria, fungus, viruses, and protozoa all play a role in this dynamic environment, potentially impacting student health. Comprehending these microbial dynamics is crucial for formulating effective public health interventions to enhance hygiene behaviors and improve overall health outcomes in educational settings.

## Microbial exposure in school settings

**Figure 6:**
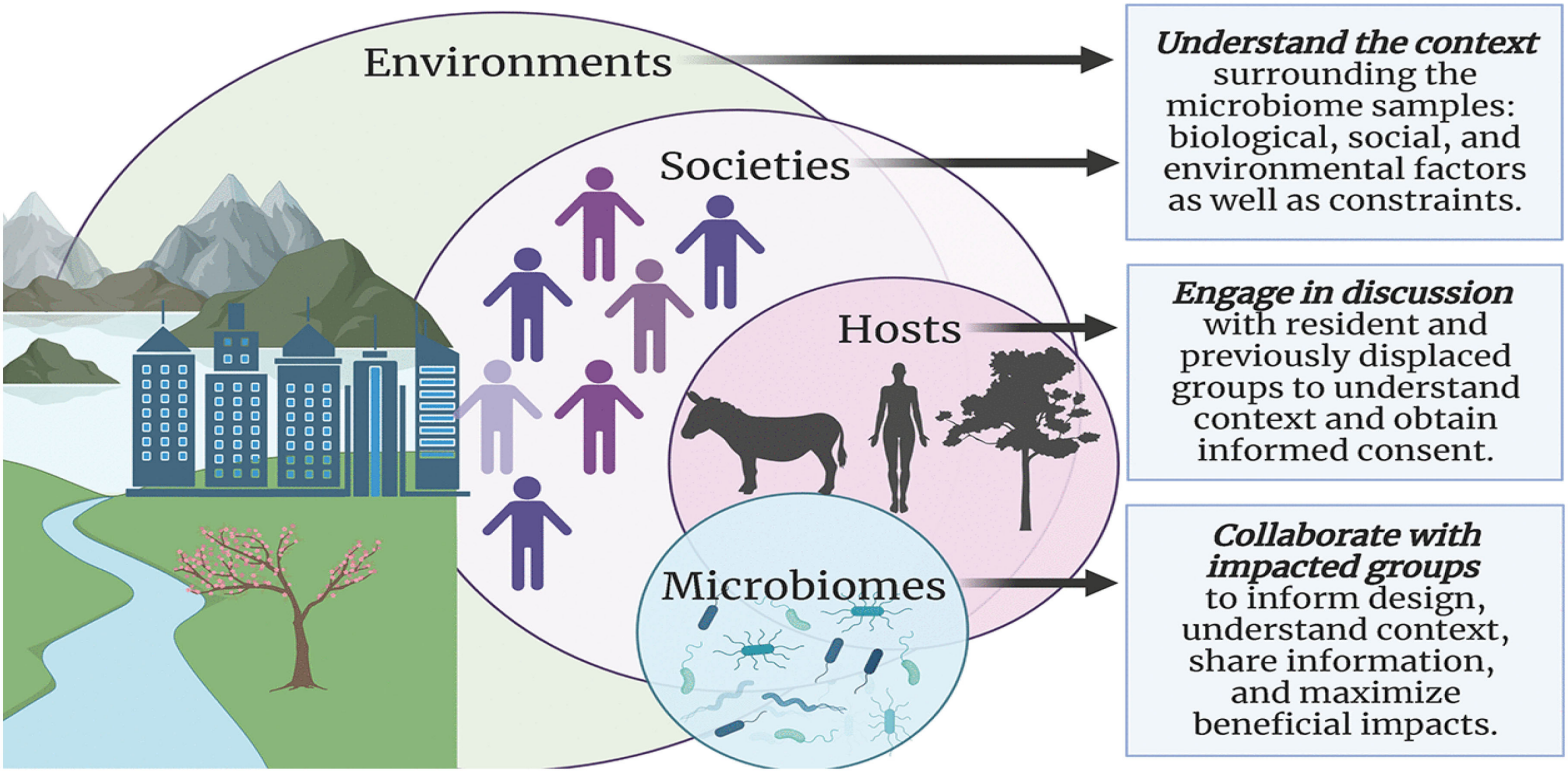
Microbial Exposure in Environmental Settings (Olvera Alvarez et al., 2018).

Karwowska’s 2003 research emphasizes the issue of microbiological air contamination in specific educational settings (**Figure 6**) (Olvera Alvarez et al., 2018). The presence of bacteria and fungi in indoor air poses a significant concern for health protection and environmental management. Accurate identification of various indoor microorganisms is crucial for assessing potential health risks and establishing quality control standards for indoor air, especially in densely populated facilities like educational institutions. This study aimed to assess the extent of microbial contamination in classrooms across kindergarten, primary school, and high school environments. The findings revealed a notably high level of microbial air pollution, surpassing both recommended microbiological standards and European Union regulatory requirements. The quantities of microorganisms, measured as CFU/m3, varied widely, ranging from 340 to 7530 for mesophilic bacteria, 5 to 35 for hemolytic bacteria, 25 to 475 for staphylococci, 0 to 45 for coli group bacteria, and 30 to 785 for molds. This study underscores the urgent need to address microbiological air contamination in educational settings.

The observed levels surpass recommended standards, underscoring the need for enhanced measures to improve indoor air quality and safeguard the well-being of occupants, particularly in densely populated educational facilities (Karwowska, 2003).

In this specific cross-sectional investigation, the researchers examined how a distinct combination of indoor air pollutants in a school setting affected the health of attending children. Specifically, issues such as a leaking roof, damp floors, and gas leaks from the sewage system resulted in a collective exposure to hydrocarbons, 2-ethylhexanol from plastic flooring, and moisture-associated microbes. The health of 274 school children was assessed through repeated surveys regarding their symptoms. Statistical analysis revealed a clear association between these indoor air pollutants and adverse health effects, including respiratory irritation, asthmatic symptoms, general discomfort, and an increased occurrence of common viral respiratory infections. However, no apparent link was found between these exposures and doctor-diagnosed asthma, other allergic conditions, or bacterial respiratory infections. The study pinpointed chemical pollutants originating from the sewage system and construction materials affected by moisture as the primary causes. Subsequent remediation of the school building led to notable improvements in both indoor air quality and the children’s health (Putus et al., 2004).

According to Kwan et al. (2018), significant attention was given to restoring microbial communities on surfaces following cleaning activities in educational settings. The primary objective was to assess changes in the indoor microbiome on school desk surfaces, particularly focusing on bacterial and fungal communities, after a cleaning intervention. The researchers employed a combination of quantitative PCR and DNA sequencing techniques to examine microbial dynamics on ten desk surfaces across three schools in the Northeastern United States. Six samples were collected from each desk: one before cleaning and five subsequent samples taken at intervals of 30 minutes, 1 day, 3 days, 7 days, and 21 days after cleaning. The cleaning process physically removed around 50% of bacteria, fungi, and human cells, and a full recovery of microbial concentrations on the surfaces occurred within a relatively short period of 2 to 5 days. This recovery time was notably faster than the schools’ established cleaning schedule, which occurred once per semester. The primary source of bacteria and fungi found on the desks during the study period was traced back to the human microbiome, including skin, oral, and gut microbiota. Additionally, it was observed that over 50% of the identified fungi on the desks belonged to genera known to contain allergenic species. The microbial communities present on these school desks predominantly originate and persist through the deposition of bacteria and fungi associated with humans. Existing cleaning procedures and schedules in schools may not effectively decrease student exposure to allergenic fungi and microbes that come from human sources (Kwan et al., 2018).

## Microbial diversity and immune system development

**Figure 7:**
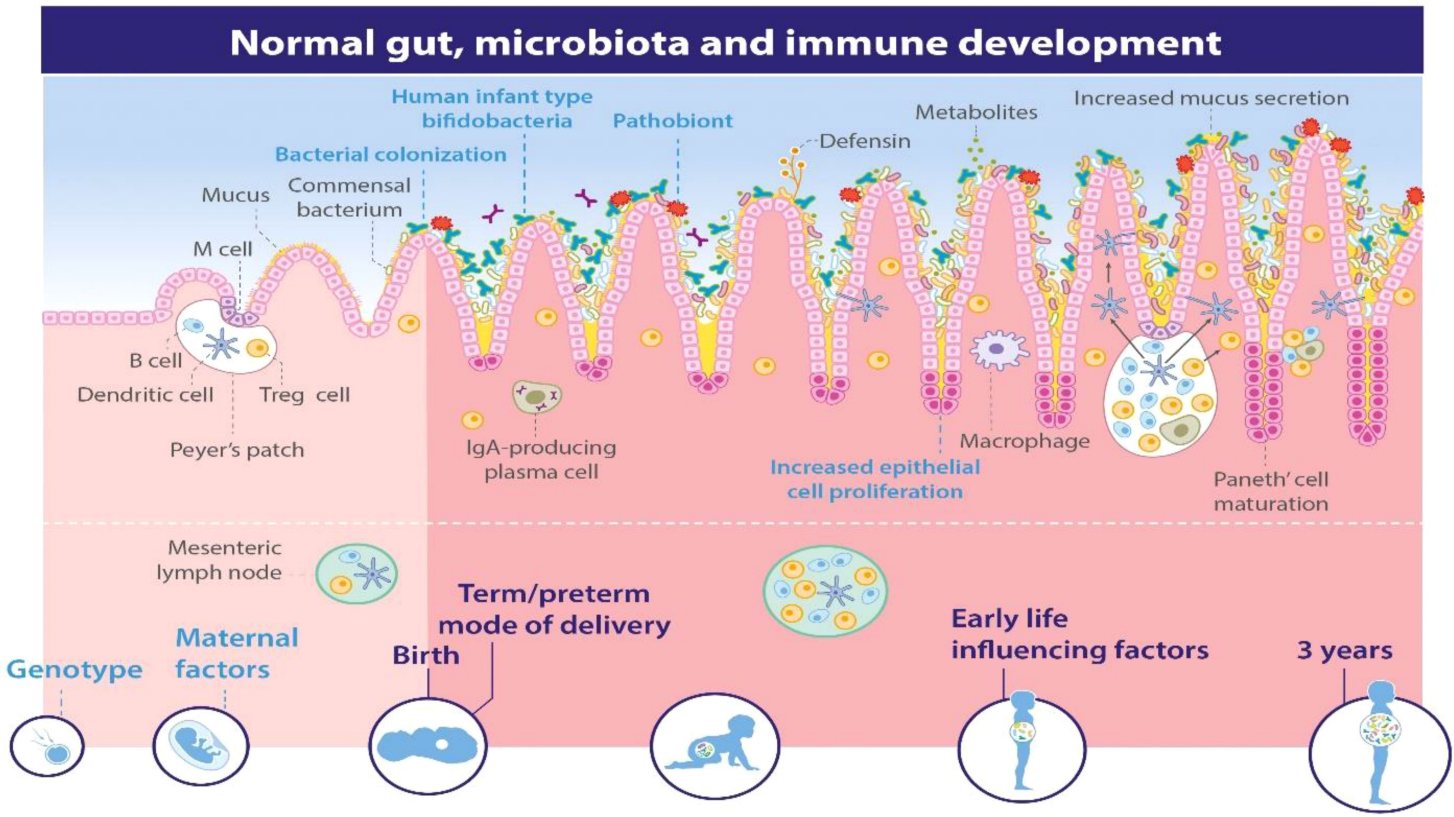
Microbial Diversity and Immune System Development (Spencer et al., 2019).

Research indicates that the mammalian digestive system hosts a diverse microbiota comprising approximately 500 to 1000 different microbial species. Studies comparing germ-free environments have highlighted the crucial role of this gut microbiota in fostering the development of both the local gut immune system and the broader systemic immune system (**Figure 7**) (Spencer et al., 2019). Early exposure to gut microbes is believed to significantly decrease the occurrence of inflammatory, autoimmune, and atopic diseases (Pierau et al., 2021). These findings emphasize the consensus in the scientific community that microbial colonization plays a vital role in regulating and refining the immune system throughout an individual’s lifespan. Recent research exploring molecular diversity has further demonstrated that the composition of the human gut microbiota undergoes alterations in individuals with inflammatory bowel disorders (Kelly et al., 2007). This indicates that certain bacterial species are crucial for preserving immune balance and general well-being. Exciting new insights are emerging regarding how gut bacteria influence the mammalian immune system. However, there is still a great deal to learn about the mechanisms through which these commensal bacteria influence the activities of both innate and adaptive immune cells in both healthy states and disease conditions (Kelly et al., 2007).

According to Bello et al. (2018), since World War II, there has been a notable rise in metabolic, immune, and cognitive disorders, including obesity, diabetes, asthma, allergies, inflammatory bowel disease, and autism. Initially, these issues were more prevalent in industrialized nations, but they are now on the rise in developing countries as well. Alongside the health impacts, the economic burden of these diseases is staggering, with obesity costing $2.0 trillion and diabetes costing $1.3 trillion globally each year. As these conditions continue to spread in developing nations, the problem is escalating rapidly. This escalating cost, both in terms of health and economies, is becoming unsustainable, as resources for caring for chronically ill adults compete with those needed for the well-being of the next generation. The question arises: are these various diseases entirely independent, or is there a mutual fundamental factor? The authors suggest that alterations in the human microbiota, coinciding with the period of industrialization, might be the underlying cause. These changes entail a loss of the microbial diversity that has been part of our evolutionary heritage for millions of years (Bello et al., 2018).

As per Mulder et al. (2009), the early establishment of gut microbial colonization is pivotal in reducing the chances of contracting infectious, inflammatory, and autoimmune diseases. Recent studies on population trends suggest that childhood hygiene practices significantly contribute to the elevated risk of developing inflammatory bowel disease (Cholapranee and Ananthakrishnan, 2016; van der Sloot et al., 2017), The hygiene hypothesis is supported and emphasizes the potential importance of microbial colonization during infancy. However, there is still much to explore regarding how the early-life environment shapes the diversity of microbes in the adult gut and the resulting immune responses. To address this inquiry, researchers utilized pigs as a model to investigate how the early-life environment influences interactions between microbes and the host gut as the animals develop. Piglets with similar genetic backgrounds were raised in various environments: indoors, outdoors, and in experimental isolators. Analysis of over 3,000 16S rRNA sequences revealed significant differences in the diversity of mucosa-adherent microbes in the ileum of adult pigs, attributable to their early-life environments. Pigs raised outdoors in natural conditions showed a prevalence of Firmicutes, particularly Lactobacillus, while those in sanitized indoor settings had lower levels of Lactobacillus and higher counts of potentially harmful microbial types. The analysis demonstrated a noticeable inverse relationship between the abundance of Firmicutes and the presence of pathogenic bacteria in the gut, especially in animals kept in experimental isolators. Using Affymetrix microarray technology and Real-time Polymerase Chain Reaction, significant gut-specific gene responses associated with the early-life environment were identified. Specifically, pigs raised indoors exhibited increased expression of Type 1 interferon genes, Major Histocompatibility Complex class I, and various chemokines. Gene Ontology and pathway analysis supported these findings. It is clear that the early-life environment has a substantial impact on both the microbial composition of the adult gut and mucosal innate immune function. The presence of a microbiota dominated by lactobacilli appears to contribute to maintaining mucosal immune balance and restricting pathogen colonization (Mulder et al., 2009).

Again, according to Cahenzli et al. (2013), the presence of microbes in the environment after birth significantly influences the development of the mammalian immune system. Changes in the composition of the microbiota have been linked to a higher occurrence of allergic and autoimmune conditions, characterized by elevated levels of a specific antibody called IgE in the blood. Previous studies have shown that mice raised in a germ-free environment, without exposure to microbes, exhibit abnormally high levels of IgE, indicating that signals from the microbiota are necessary to regulate normal IgE levels. Our research demonstrates that both germ-free mice and those with a limited variety of microbes experience heightened levels of IgE in early life. In neonatal germ-free mice, B cells in mucosal areas undergo a process called isotype switching to produce IgE, and this is influenced by CD4 T-cells and a molecule called IL-4. It has been found that a certain level of microbial diversity after birth is crucial to prevent the overproduction of IgE. Elevated IgE levels in germ-free mice result in an increase in IgE bound to the surface of mast cells, leading to an exaggerated systemic allergic reaction triggered by oral exposure to allergens. This underscores the significance of appropriate microbial stimuli in the intestines during early life in establishing a regulatory network that guards against excessive IgE production in mucosal areas (Cahenzli et al., 2013).

## Hygiene practices in school

In developing countries, a significant portion of illness and death is attributed to infectious diseases, accounting for 31% of all deaths in Southeast Asia. Inadequate awareness of the health benefits of personal hygiene contributes to poor health outcomes in school children (Meher and Nimonkar, 2018). This recent six-month cross-sectional study was conducted among 440 students attending a government school in Kolkata, West Bengal. The aim was to evaluate the hygiene habits of these students. The majority of children reported having access to clean water both at home (94%) and at school (84%). While the overall percentage of good hygiene practices among the students was deemed satisfactory, when asked to demonstrate the correct hand-washing procedure, 86.1% performed it incorrectly (Meher and Nimonkar, 2018).

According to Nair et al. (2012) a study was conducted and the aim of this study was to investigate menstrual issues and hygiene practices among adolescent girls in Thiruvananthapuram City Corporation. The participants were selected from ten Higher Secondary Schools in the area, consisting of students in class XI and XII aged between 15 to 19 years. A multistage sampling procedure was used, and the students were assessed using a pretested self-evaluation questionnaire. The study found that 21.1% of the girls reported experiencing menstrual disorders. The most frequently cited problem during menstruation was dysmenorrhea (72.4%), followed by oligomenorrhoea (11.3%). Only 11.5% of the girls with menstrual issues sought treatment, with the majority seeking help from gynecologists. Among the girls who reported vaginal discharge (81.5%), only 5.7% had abnormal discharge. Overall, the majority of the girls demonstrated adequate menstrual hygiene. This study underscores the prevalence of menstrual disorders among adolescent girls, highlighting the potential impact on their future reproductive health (Nair et al., 2012).

**Figure 8:**
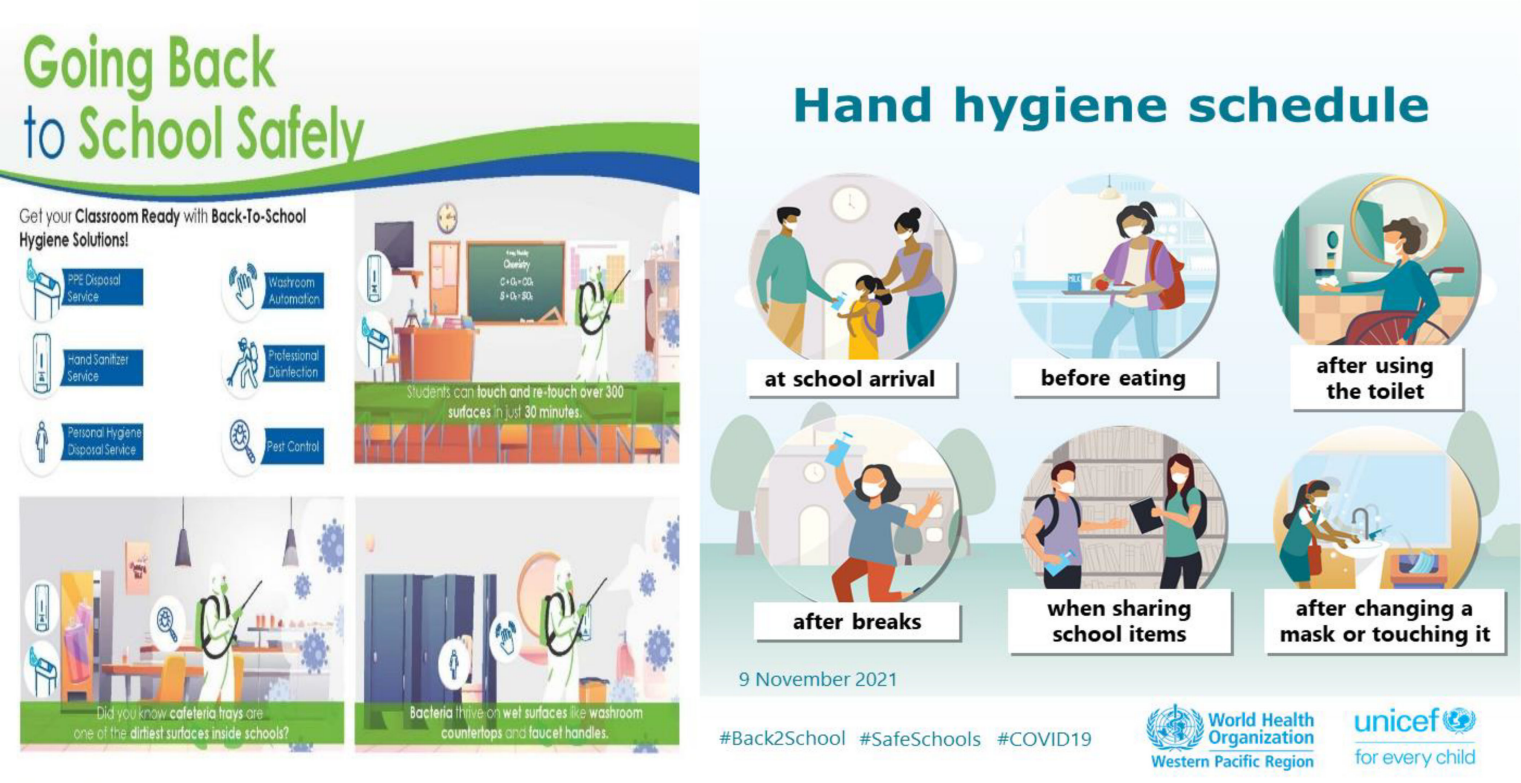
Personal hygiene practices and education (Allegranzi et al., 2022; Dewan et al., 2022).

Thakadu et al. (2018) suggest that improving sanitation and personal hygiene practices is crucial for reducing the spread of communicable diseases and enhancing public health. Diarrheal-related deaths, especially among adolescents aged 10-19, are among the leading causes of mortality globally, with the 10-14 age group being particularly affected. Primary school students in developing nations are especially vulnerable. Addressing personal hygiene among school children not only reduces mortality and illness but also leads to improved school attendance and better learning outcomes (**Figure 8**). Thus, it is essential to effectively address water, sanitation, and hygiene issues within the school environment to promote better health and education. This study examined hygiene education, personal hygiene practices, and environmental sanitation in three primary schools in Botswana’s Ngamiland district. Using proportionate stratified random sampling, 285 students and 15 teachers were selected as key informants. Data was collected through semi-structured questionnaires for key informants and a social survey instrument for students. The findings revealed that a small percentage of students associated poor hygiene with diseases such as diarrhea/upset stomach (31.7%), malaria (23%), bilharzia (16.4%), and cholera (14.8%), indicating a limited understanding of hygiene. Hygiene education is integrated into the curriculum, and teacher training in this area is mainly provided through in-service workshops. Concerning personal hygiene practices, over 70% of students reported “always” washing their hands before and after eating, while slightly over one-fifth indicated “sometimes.” The majority of students dispose of solid waste properly in designated containers, with very few littering on school premises. The low levels of hygiene knowledge observed among students in the study area could negatively impact their academic performance and make them susceptible to infectious diseases, leading to absenteeism. This highlights the need to implement initiatives beyond the current educational approach, such as establishing extracurricular clubs to promote pro-hygiene behaviors and engage adolescents in meaningful and sustained participation (Thakadu et al., 2018).

## The hygiene hypothesis and immune function

In the realm of immunology, the Hygiene Hypothesis stands as a fascinating and thought-provoking theory that challenges conventional wisdom about the role of cleanliness in shaping immune function (Wilson et al., 2020). This hypothesis proposes that reduced exposure to infectious agents and microbes in early childhood may contribute to an augmented susceptibility to allergies and autoimmune diseases later in life. As I delved into the intricate web of research surrounding this concept, I found myself captivated by the intricate dance between hygiene and immune response (Chatterjee and Noble, 2016). The cornerstone of the Hygiene Hypothesis lies in its assertion that a lack of microbial exposure during childhood may hinder the development of a robust immune system. Research investigations have explored the microbial interactions occurring in infancy, highlighting their pivotal role in educating the immune system to differentiate between benign and detrimental substances (National Academies of Sciences & Medicine, 2017; Netea et al., 2011; Yeşilyurt et al., 2021). This perspective challenges the prevailing notion that stringent cleanliness is always beneficial, suggesting that a certain degree of microbial exposure might be essential for immune system maturation. One of the most compelling aspects of the Hygiene Hypothesis is its potential to explain the rising prevalence of allergies and autoimmune disorders in industrialized societies (Lambrecht and Hammad, 2017). As our societies become more obsessed with cleanliness and sanitized environments, the hypothesis postulates that we may be inadvertently compromising our immune system’s aptitude to function optimally. The idea that a lack of early exposure to diverse microbes could lead to immune dysfunction opens up new avenues for understanding and potentially preventing these modern health challenges. However, the Hygiene Hypothesis is not without its skeptics. Critics argue that it oversimplifies the complex interplay between genetics, environment, and immune function (Hagen, 2015). They point out that while microbial exposure is undoubtedly a factor, it is not the sole determinant of immune health. Moreover, they caution against promoting unhygienic practices in an attempt to boost immune function, as this could pose serious health risks. Despite the debates surrounding the Hygiene Hypothesis, it undeniably serves as a catalyst for reevaluating our approach to hygiene and immune health. Researchers continue to unravel the intricate mechanisms underlying this phenomenon, shedding light on the nuances of microbial interactions and their profound impact on our immune systems (Hooks et al., 2019; Koskella et al., 2017; Smith et al., 2016). The Hygiene Hypothesis stands as a reminder that, in our quest for cleanliness, we must strike a delicate balance that fosters a resilient immune system without compromising overall health. As the scientific community grapples with the complexities of this hypothesis, it offers a captivating journey into the evolving understanding of the intricate relationship between hygiene and immune function.

Bach (2018) highlights a concerning trend – the consistent rise in autoimmune diseases alongside a parallel decline in most infectious diseases. This juxtaposition has given rise to the hygiene hypothesis, suggesting that the reduced incidence of infections directly contributes to the increased prevalence of autoimmune and allergic diseases. While epidemiological data strongly supports this hypothesis, the specific mechanisms at play remain unclear. Notably, various pathogens, including bacteria, viruses, and parasites, have been shown to prevent autoimmune diseases in experimental models. Additionally, the role of gut commensal bacteria is crucial, as observed dysbiosis in autoimmune disease patients suggests a connection, although causality remains uncertain. Both pathogens and commensals impact immunoregulatory pathways, with a particular emphasis on the involvement of innate immune receptors, notably Toll-like receptors, in mediating the protective effects against autoimmunity (Bach, 2018).

**Figure 9:**
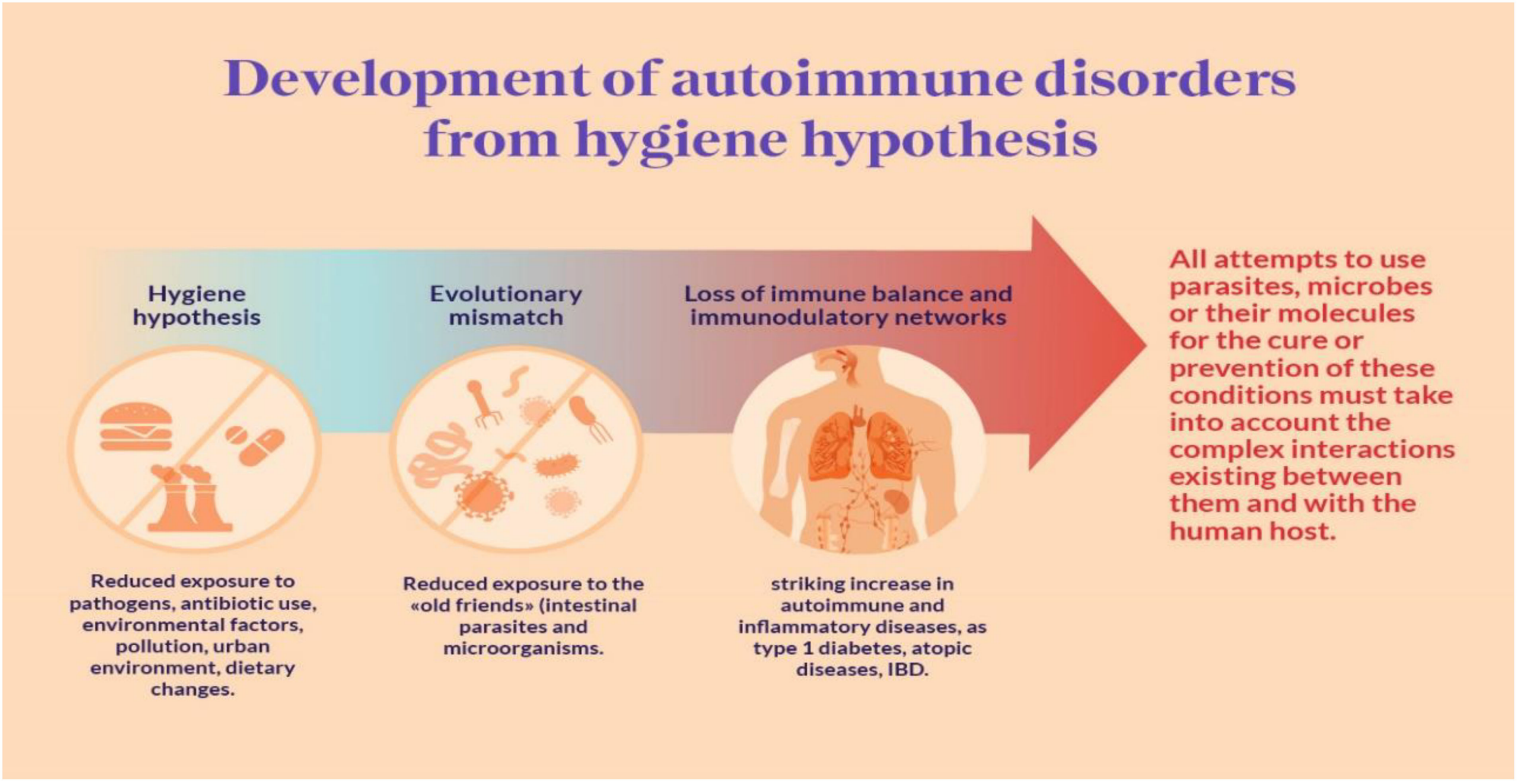
Hygiene Autoimmune (Elsawey et al., 2022).

Stiemsma et al. (2015) suggest that developed nations have experienced a steady increase in atopic diseases and immune dysregulation since the 1980s, coinciding with a decrease in infectious diseases during the same timeframe. Conversely, developing countries show an opposite trend, with lower rates of immune dysregulation and a higher incidence of infectious diseases. Initially introduced by Strachan in 1989, the “hygiene hypothesis” was formulated to account for this phenomenon (**Figure 9**). However, recent research spanning the last decade has extended this hypothesis to include the role of commensal and symbiotic microbes, specifically the intestinal microbiota, and parasitic helminths in immune development, evolving into the “microflora” and “old friends” hypotheses, respectively (Liddicoat et al., 2016; Lloyd-Price et al., 2016). Research findings indicate that parasitic helminths and symbiotic microbial organisms have evolved alongside the human immune system, contributing significantly to fostering normal immune development. Recent studies highlight the prospect of modifying the bacterial gut microbiota to address and potentially prevent immune dysregulation, including atopic diseases and other immune-related conditions such as inflammatory bowel disease and type 1 diabetes (Paun and Danska, 2016; Paun et al., 2016). Both human and animal model studies are essential for understanding the mechanistic connections between intestinal microbes, helminth parasites, and the human immune system. Various therapeutic strategies are being investigated, including pro-, pre-, and symbiotic therapies, as well as interventions utilizing live helminths and their excretory/secretory products. These approaches show potential for addressing and preventing diseases linked to immune dysregulation. Moving forward, personalized treatments involving microbiota and/or helminth interventions administered early in life are expected to have a significant impact on reducing the incidence of inflammatory bowel disease, type 1 diabetes, and atopic disorders (Stiemsma et al., 2015).

## Model describing the role of beneficial or pathogenic microorganisms in affecting public health in school settings

**Figure 10:**
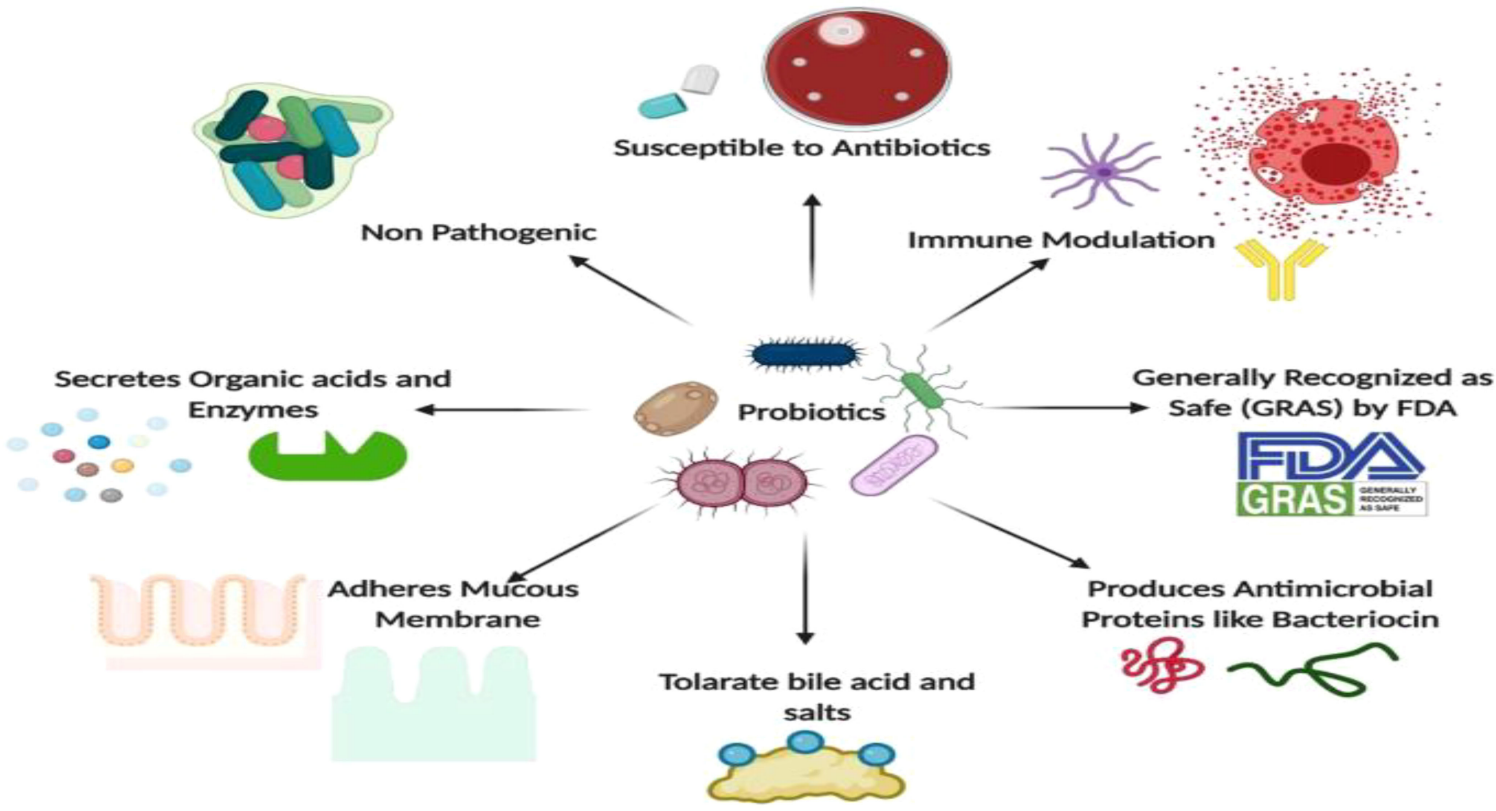
Applications of Microbes in Human Health ( (Jasmine et al., 2022).

Microorganisms play a crucial role in human health, primarily through their interactions with the human microbiome. These beneficial microbes, including probiotics, contribute to various health benefits, such as enhancing immune function, preventing diseases, and promoting overall well-being (**Figure 10**) (Jasmine et al., 2022). The understanding of these interactions is vital for developing personalized healthcare strategies and leveraging microbial applications in medicine.

Probiotics, such as *Lactobacillus* and *Bifidobacterium*, are known for their anti-inflammatory, antimicrobial, and immunomodulatory properties (Jasmine et al., 2022). They help maintain gut health by competing with pathogenic bacteria and enhancing the immune response (Upadhyaya et al., 2016). Moreover, disruption of the microbiome can lead to autoimmune diseases, gastrointestinal cancers, and metabolic disorders (Elshafei, 2021).

Research indicated that restoring microbial balance through probiotics can mitigate these health issues (Elshafei, 2021). Microorganisms also serve as a source of single-cell protein, providing essential nutrients and vitamins, which can be beneficial in addressing global food shortages (Upadhyaya et al., 2016).

## Microbial intervention strategies in school settings

**Figure 11:**
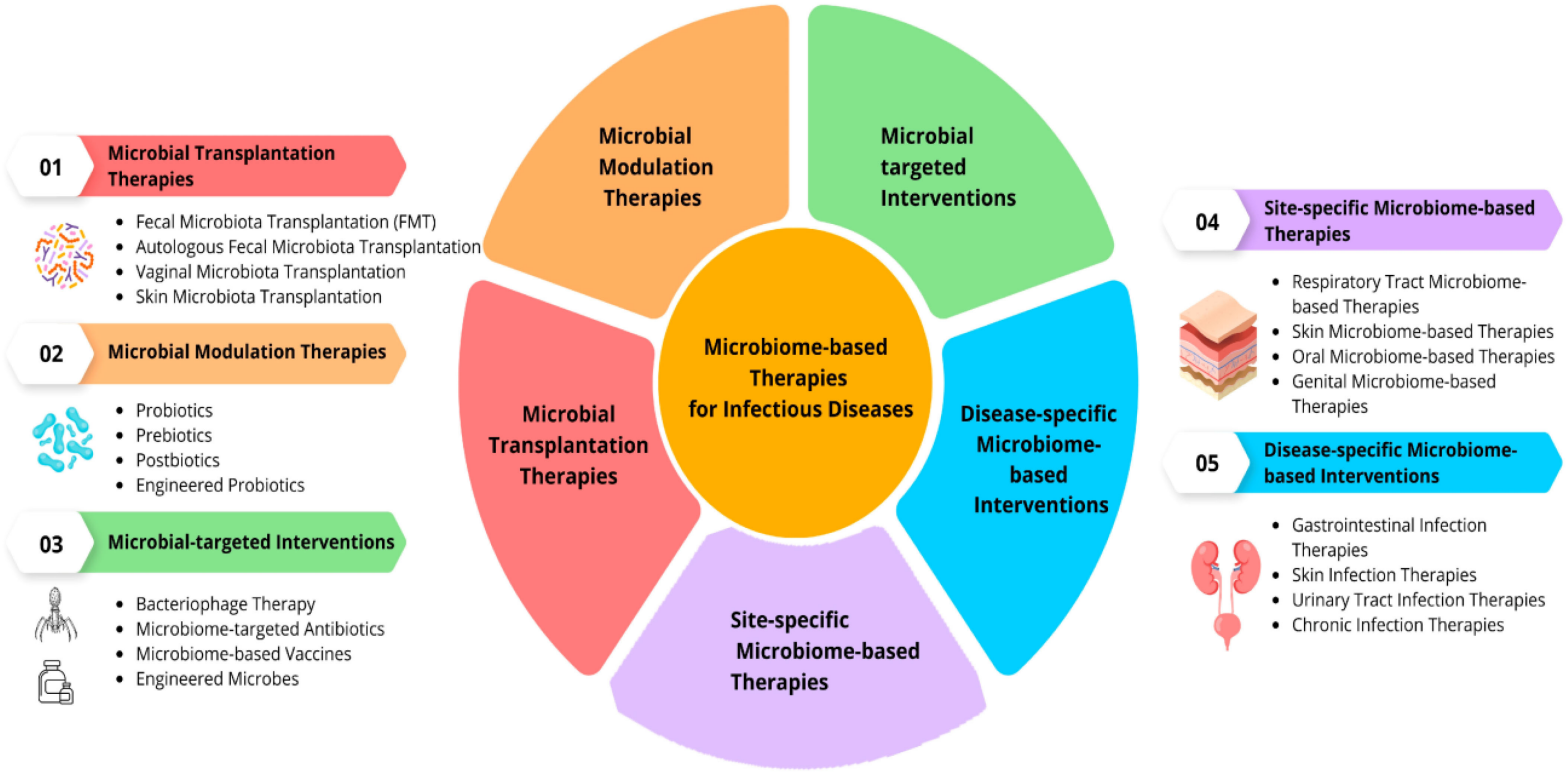
Microbial Intervention Strategies (Mukherjee et al., 2018).

Microbial intervention strategies refer to the deliberate use of microorganisms to positively influence biological systems, ecosystems, or industrial processes (Peralta et al., 2014). These strategies harness the unique capabilities of microbes for various applications, ranging from improving human health to environmental remediation and industrial production. The use of microbial intervention has gained prominence due to advancements in microbiology, genetic engineering, and our growing understanding of microbial ecosystems (Maurya et al., 2021). In the realm of human health, microbial intervention strategies have made significant strides (**Figure 11**) (Mukherjee et al., 2018). Probiotics, for example, involve the administration of beneficial bacteria to the digestive system, promoting a balanced microbial community in the gut (Sánchez et al., 2017).

Educational interventions centered around microbiology can significantly enhance students’ understanding of microorganisms and their impact on health. A study highlighted the development of microbiological learning resources tailored for schools, which emphasized practical activities linked to the curriculum (Scalas et al., 2017). This approach not only engaged students but also addressed gaps in practical microbiology education, where many teachers reported a lack of resources and confidence in delivering such content.

Service-learning initiatives have emerged as effective strategies for integrating microbiology education into school settings. A recent study demonstrated how university students engaged primary school pupils through hands-on activities that illustrated the dual role of microbes both beneficial and harmful (Rollwagen-Bollens et al., 2022). This method not only improved scientific literacy but also instilled a sense of responsibility regarding hygiene practices and microbial awareness among young learners (Shabangu, 2023).

Given the rising concern over antibiotic resistance, educational programs focused on proper antibiotic use are crucial. A study conducted in Portugal assessed the impact of targeted teaching interventions on middle school students’ knowledge regarding antibiotics. The results showed significant improvements in understanding appropriate antibiotic use and the risks associated with misuse, highlighting the potential of school-based interventions to influence public health positively (Shabangu, 2023).

Integrating microbiology into existing school curricula is vital for fostering an informed student body. The “Tiny Earth” initiative exemplifies how educators can engage students in real-world microbiology through project-based learning focused on antibiotic discovery (Penprase, 2020). Such programs not only teach scientific concepts but also encourage students to contribute to public health discussions surrounding antimicrobial resistance (Gordy et al., 2021).

## Impact on allergies and autoimmune diseases

In recent years, there has been increasing acknowledgment of the impact of microbial interactions on human health, especially within school environments (De Giani et al., 2021). Microbes play a critical role in shaping the immune system and its response to allergens and autoimmunity.

Numerous studies have indicated that a decrease in microbial diversity is associated with a higher likelihood of developing allergies. The “hygiene hypothesis” proposes that early-life exposure to diverse microbial communities helps educate and regulate the immune system, thereby reducing the likelihood of developing allergies (Perkin and Strachan, 2022). In school settings, the abundance and diversity of microbial communities can vary, influenced by factors such as cleaning practices, ventilation systems, and social interactions among students (Mills et al., 2023). It has been suggested that exposure to a wide range of beneficial microbes, including those found in natural environments, may have a protective effect against allergies (Renz and Skevaki, 2021). Specific examples of microorganisms that have been studied for their health impacts include various strains of *Lactobacillus*, *Bifidobacterium*, and environmental fungi such as *Penicillium* and *Aspergillus* (**Table 1**). These microorganisms can either positively influence health by enhancing immune responses or negatively impact health through allergenic or pathogenic mechanisms.

**Table 1:** Microorganisms impacting health in school settings.

Microorganism	Type	Health Impact	Notes
*Lactobacillus* spp.	Beneficial	Enhances gut health, supports immune function	Commonly found in fermented foods
*Bifidobacterium* spp.	Beneficial	Promotes digestive health, may reduce allergies	Present in breast milk and probiotics
*Penicillium* spp.	Potentially Harmful	Allergic reactions, respiratory issues	High indoor concentrations linked to asthma
*Aspergillus* spp.	Potentially Harmful	Allergic fungal sinusitis, respiratory infections	Common in damp environments
*Staphylococcus aureus*	Pathogenic	Skin infections, respiratory infections	Often found on surfaces in schools
*Escherichia coli*	Pathogenic	Gastrointestinal illnesses	Contamination from poor hygiene practices
*Klebsiella pneumoniae*	Pathogenic	Respiratory infections, urinary tract infections	Found on contaminated surfaces

The presence of both beneficial and harmful microorganisms within school environments can significantly influence student health. For instance, beneficial microbes like *Lactobacillus* and *Bifidobacterium* are known to enhance gut microbiota diversity and support immune function, potentially reducing the risk of allergic diseases (Fang et al., 2021). Conversely, exposure to high levels of allergens from fungi such as *Penicillium* and *Aspergillus* can exacerbate asthma and other respiratory conditions among children (Hadebe and Brombacher, 2019). Moreover, pathogenic bacteria like *Staphylococcus aureus* and *Escherichia coli* pose significant risks due to their association with common infections that can lead to increased absenteeism in schools. Understanding the balance between these microorganisms is crucial for developing effective public health strategies aimed at improving indoor air quality and hygiene practices within educational settings.

**Figure 12:**
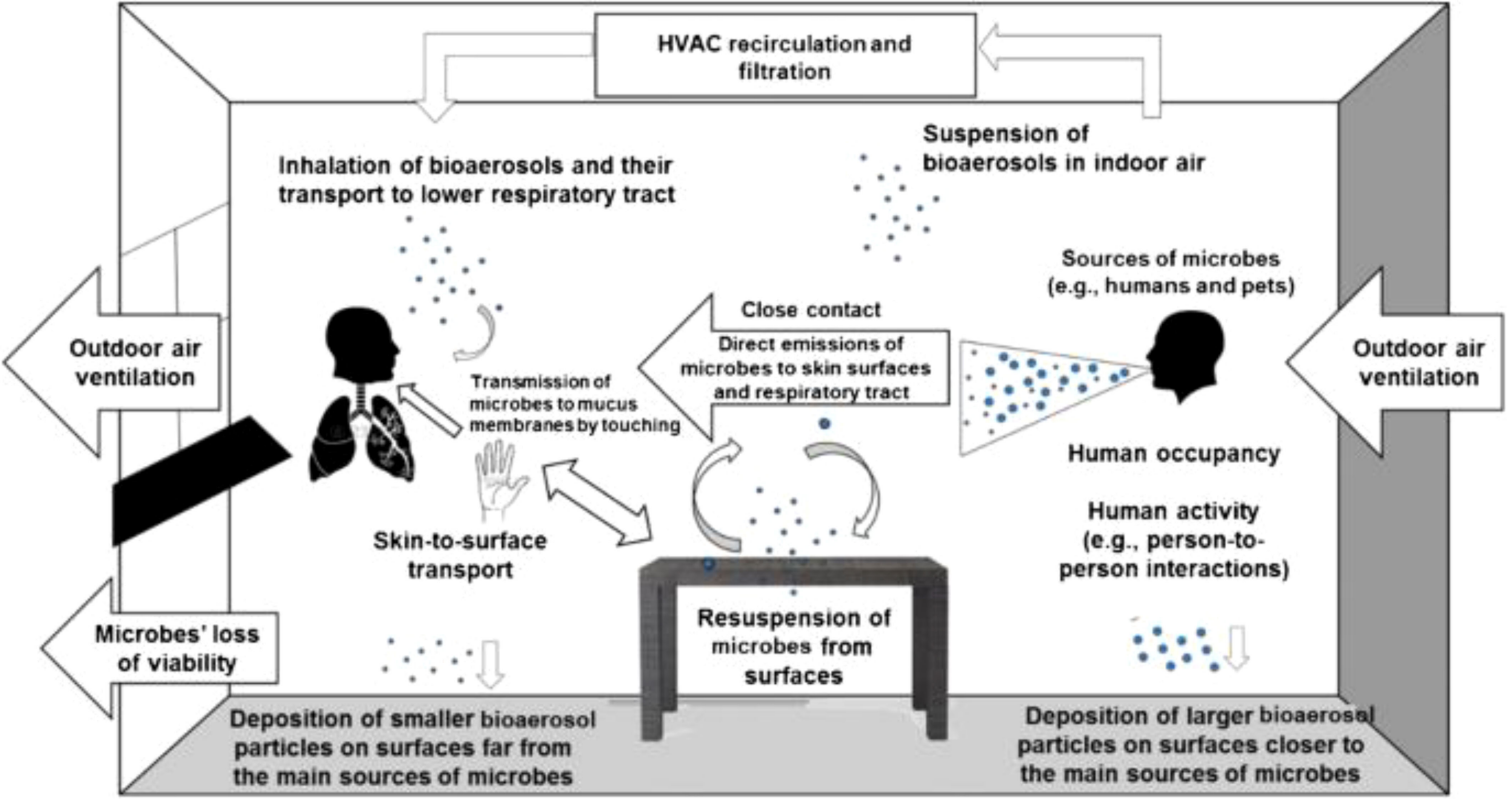
School Microbial Exchange (Stephens et al., 2019).

The gut microbiome plays a vital role in autoimmune diseases by modulating immune responses and maintaining immune tolerance (**Figure 12**). Research indicates that autoimmune diseases, especially autoimmune neuroinflammation and systemic lupus erythematosus (SLE), are closely linked to alterations in gut microbial composition (Steimle et al., 2023; Wang et al., 2024; Yao et al., 2023). Dysbiosis in the gut microbiota can lead to immune dysregulation, promoting autoimmunity (Surapsari et al., 2023). Studies have shown that specific microbial signatures are associated with autoimmune diseases, such as the enrichment of certain genera like Enterococcus and Lactobacillus in autoimmune diseases like SLE (Hasimoto, 2023). Furthermore, dysbiosis can trigger autoimmunity by influencing thymic selection thresholds of self-reactive T cells and microbiota-reactive T cells, ultimately leading to immune dysregulation and disease development. Therefore, understanding and targeting the gut microbiome may offer potential therapeutic strategies for managing autoimmune diseases. School settings, with their shared spaces and interactions, provide opportunities for microbial exchange among students, potentially influencing the development of autoimmune diseases.

Understanding the microbial dynamics in school settings can inform interventions and strategies to promote health and immune enhancement. Probiotics and prebiotics have been investigated as potential interventions to modulate the gut microbiome and reduce the risk of allergies and autoimmune diseases (Bai et al., 2024; Dargahi et al., 2019). Additionally, implementing measures to promote microbial diversity, such as exposure to natural environments, optimizing ventilation systems, and encouraging outdoor activities, may help support a healthy immune system (Robinson et al., 2022).

## Mitigating the risk of pathogen transmission

**Figure 13:**
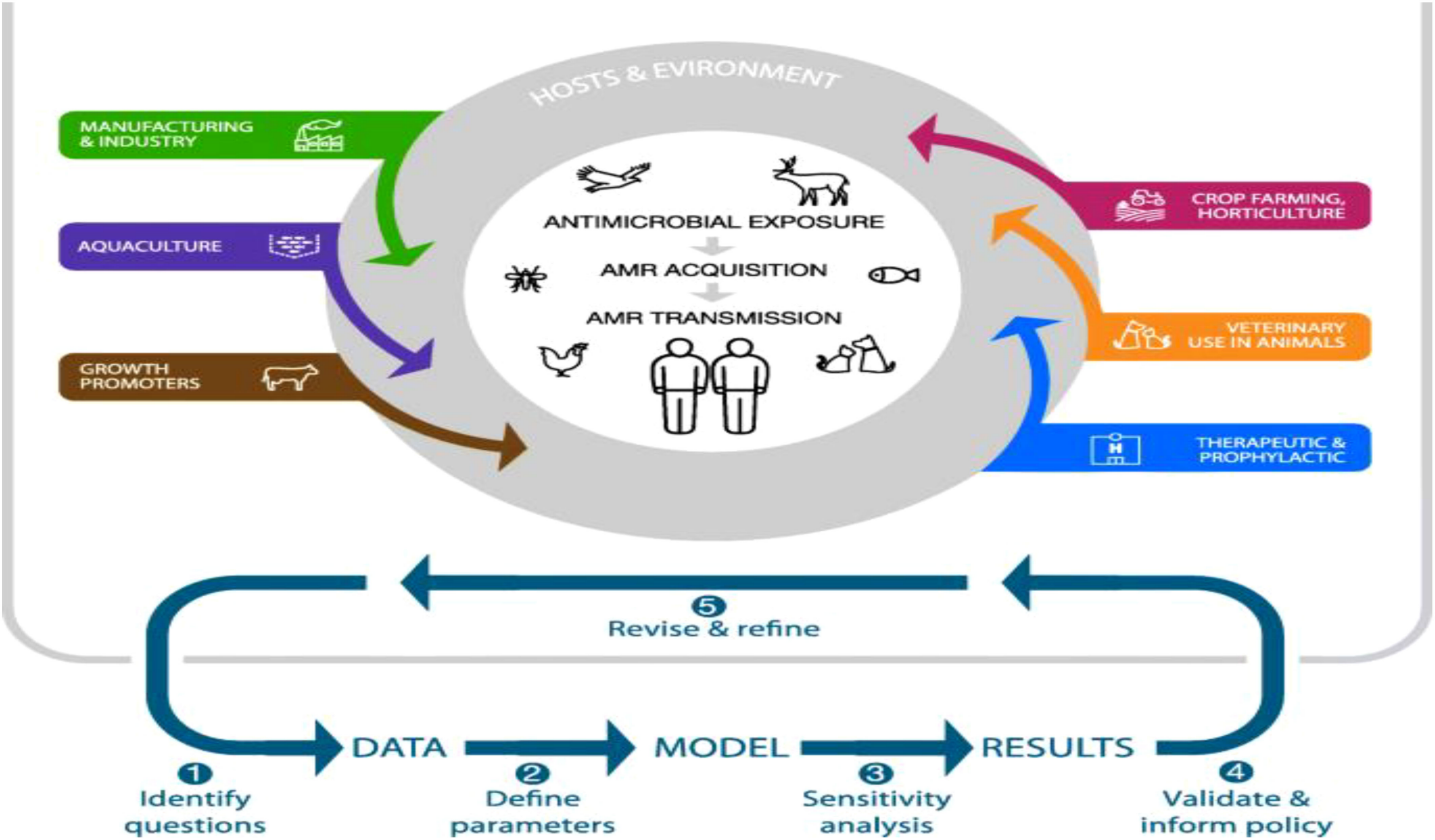
Epidemiological Modeling of Pathogen Spread (Niewiadomska et al., 2019).

In recent years, the global community has witnessed the devastating consequences of infectious disease outbreaks, underscoring the urgent need for robust strategies to mitigate pathogen transmission (Nii-Trebi, 2017). The emergence of new pathogens, such as SARS-CoV-2, Ebola virus, and multidrug-resistant bacteria, has highlighted the interconnectedness of health systems and the vulnerability of populations to infectious threats. As a result, endeavors to reduce the transmission of pathogens have become a focal point of public health initiatives globally (Madhav et al., 2018). One of the fundamental pillars of pathogen transmission mitigation is the promotion of preventive measures at both individual and population levels. These measures include adherence to proper hand hygiene practices, respiratory etiquette, and vaccination against vaccine-preventable diseases. Additionally, environmental sanitation, effective waste management, and food safety protocols play pivotal roles in reducing the transmission of pathogens in various settings, from healthcare facilities to community environments. Furthermore, the integration of advanced surveillance systems, rapid diagnostic technologies, and real-time data analytics enables early detection and response to infectious disease threats (Zhang et al., 2023). Timely identification of outbreaks facilitates the implementation of targeted containment measures, including quarantine, contact tracing, and social distancing, which are instrumental in limiting the spread of pathogens and mitigating the impact on public health infrastructure (**Figure 13**) (Niewiadomska et al., 2019). In parallel, the dissemination of accurate, science-based information and the promotion of risk communication strategies are essential components of effective pathogen transmission mitigation efforts (Authority et al., 2021). Clear communication channels between public health authorities, healthcare providers, policymakers, and the general public foster transparency, trust, and compliance with recommended interventions, thereby enhancing community resilience and reducing misinformation-driven behaviors that may exacerbate transmission risks. As the global landscape of infectious diseases continues to evolve, it is imperative to prioritize investments in research, innovation, and capacity building to strengthen preparedness and response capabilities (Bloom and Cadarette, 2019). Multisectoral collaboration, resource mobilization, and cross-border cooperation are essential for addressing the multifaceted challenges posed by emerging pathogens and endemic diseases, ensuring equitable access to healthcare services, and safeguarding the well-being of vulnerable populations. In conclusion, mitigating the risk of pathogen transmission is an ongoing imperative that demands sustained commitment, vigilance, and adaptability from governments, healthcare systems, civil society organizations, and individuals worldwide. By embracing a holistic and proactive approach to infectious disease control, societies can mitigate the impact of pandemics, reduce health disparities, and build resilient communities capable of navigating future public health crises with confidence and solidarity.

**Figure 14:**
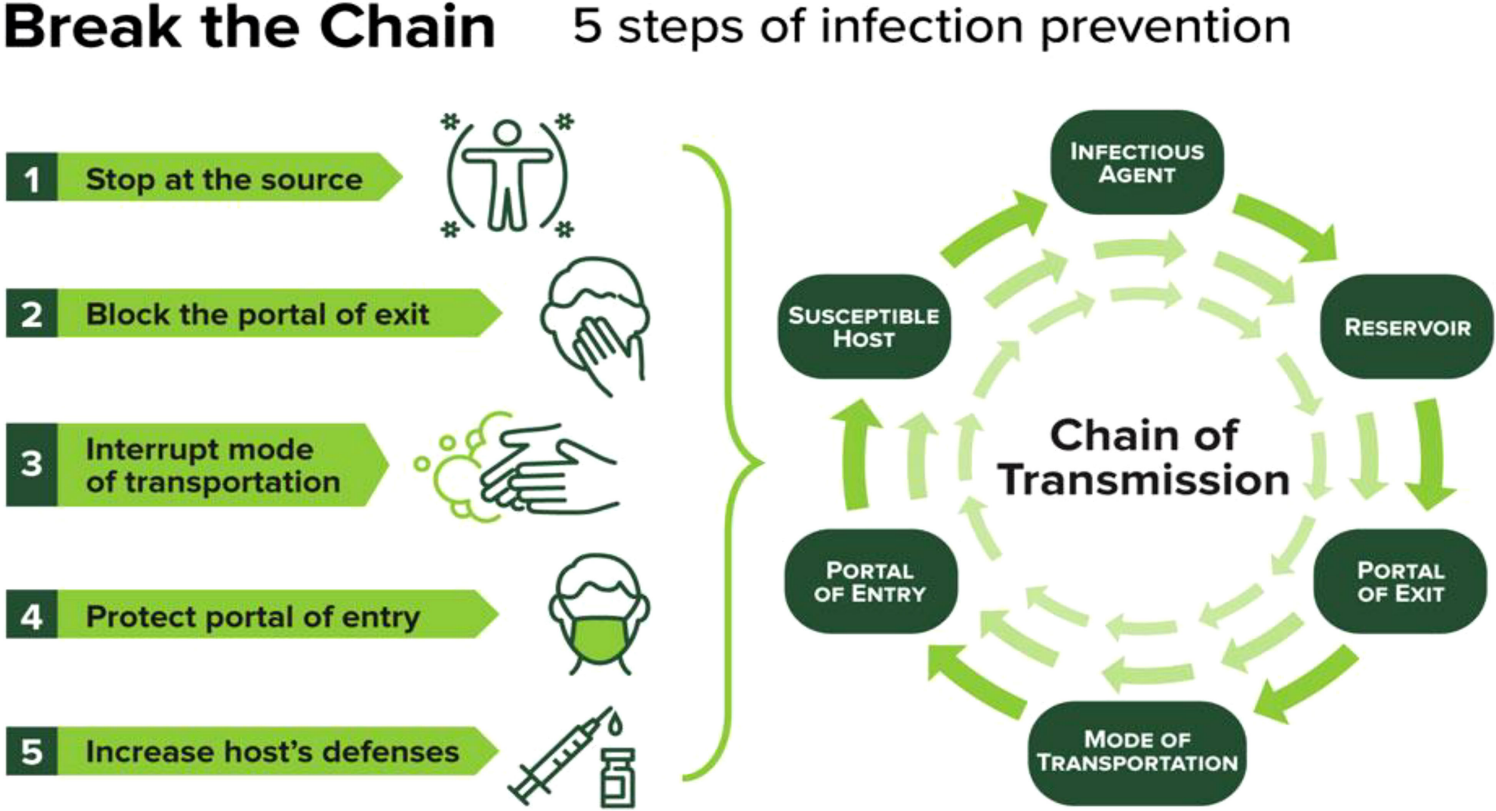
Pathogen Transmission Mitigation Infographic (Oren and Brown, 2022).

Schools play a critical role in promoting preventive measures at both individual and community levels. Implementing proper hand hygiene practices, respiratory etiquette, and vaccination against vaccine-preventable diseases are fundamental strategies. Educational programs can enhance students’ understanding of these practices, thereby fostering a culture of health within the school community (Greenberg et al., 2017). Effective sanitation protocols are essential for reducing pathogen transmission in schools. Regular cleaning and disinfection of high-touch surfaces, coupled with proper waste management and food safety protocols, can significantly lower the risk of outbreaks (Yong and Calautit, 2023). Improving indoor air quality through enhanced ventilation is vital in mitigating airborne pathogens. Studies have shown that natural ventilation or a combination of mechanical and natural ventilation can effectively reduce virus transmission in classrooms (Fageha and Alaidroos, 2022). Schools should consider implementing these strategies to create a safer indoor environment for students and staff (**Figure 14**) (Oren and Brown, 2022).

Furthermore, cohorting involves grouping students to minimize interactions between different groups, thereby reducing the potential spread of pathogens (Ismail et al., 2021). Additionally, physical distancing measures can be adapted based on local epidemiological data to further limit exposure during times of elevated illness activity (Lucchini et al., 2021). Establishing systems for illness monitoring and clear communication channels between school authorities, public health officials, and families is crucial. Timely notification of potential exposures to communicable diseases allows for prompt action and helps maintain a safe school environment (Adams, 2017).

While the focus remains on school settings, it is important to recognize that effective pathogen transmission mitigation strategies can have broader implications for public health. Schools serve as microcosms of communities; therefore, successful interventions can extend beyond educational institutions to influence family practices and community health overall.

## Future implications and research directions

This thorough review provides insights into several crucial aspects, such as the effectiveness of probiotics and microbiome interventions, the influence of hygiene practices on microbial diversity, and the significance of early microbial exposure in immune development among children of school age. By delving into these topics, the review identifies key research directions and future implications for promoting student health and immune resilience in school settings. These directions are as follows; Probiotics and Microbiome Interventions are ripe for investigation within school settings. Research could explore the efficacy of introducing beneficial microbes through probiotics or dietary supplements to enhance student health and immune function. Longitudinal studies tracking microbial changes and their effects over time would be valuable. The relationship between Hygiene Practices and Microbial Diversity warrants exploration. Identifying optimal hygiene protocols that balance disease prevention with preserving beneficial microbial communities is crucial. Research should assess environmental factors like cleaning products and ventilation systems in shaping microbial dynamics within schools. Understanding Immune Development and Early Exposure among school-aged children is essential. Long-term studies tracking immune profiles of students exposed to diverse microbial environments can shed light on immune tolerance and susceptibility to diseases later in life. Insights into the mechanisms underlying microbial exposure and immune maturation are crucial for promoting immune resilience. The Impact of Indoor Environments on Microbial Communities needs further investigation. Factors like building design, air quality, and green spaces significantly influence microbial diversity and transmission dynamics. Evaluating interventions such as indoor plants and air filtration systems is key to promoting a healthy indoor microbiome. Promoting Microbial Literacy through educational interventions can profoundly impact public health outcomes. Evaluating the effectiveness of interventions aimed at enhancing microbial literacy and hygiene practices among students, teachers, and school staff is critical. Assessing long-term behavioral changes resulting from microbial education programs is a promising research avenue. lastly, unraveling microbial dynamics in school settings offers numerous research opportunities with far-reaching implications for public health and education. By integrating microbiology, immunology, public health, and education, researchers can develop evidence-based strategies to promote a healthy microbial environment and enhance immune resilience in schools.

## Conclusion

The exploration of microbial dynamics within school settings unveils significant insights into fostering student health and immune enhancement. Through a comprehensive review of probiotics, hygiene practices, and early microbial exposure, this study underscores the importance of promoting a balanced microbial environment to support immune resilience among school-aged children. As we further explore the intricacies of microbial interactions within educational environments, it becomes increasingly evident that targeted interventions and educational initiatives can play a pivotal role in shaping student health outcomes. By integrating evidence-based strategies and advancing our knowledge of microbial dynamics, we can pave the way for healthier school environments and empower future generations with the tools they need to thrive.
